# GABAergic neuron-to-glioma synapses in diffuse midline gliomas

**DOI:** 10.1038/s41586-024-08579-3

**Published:** 2025-02-19

**Authors:** Tara Barron, Belgin Yalçın, Minhui Su, Youkyeong Gloria Byun, Avishai Gavish, Kiarash Shamardani, Haojun Xu, Lijun Ni, Neeraj Soni, Vilina Mehta, Samin Maleki Jahan, Yoon Seok Kim, Kathryn R. Taylor, Michael B. Keough, Michael A. Quezada, Anna C. Geraghty, Rebecca Mancusi, Linh Thuy Vo, Enrique Herrera Castañeda, Pamelyn J. Woo, Claudia K. Petritsch, Hannes Vogel, Kai Kaila, Michelle Monje

**Affiliations:** 1 Department of Neurology and Neurological Sciences, Stanford University, Stanford, CA USA; 2 Howard Hughes Medical Institute, Stanford University, Stanford, CA USA; 3 Department of Neurosurgery, Stanford University, Stanford, CA USA; 4 Department of Pathology, Stanford University, Stanford, CA USA; 5 Faculty of Bio- and Environmental Sciences (MIBS), University of Helsinki, Helsinki, Finland; 6 Neuroscience Center (HiLIFE), University of Helsinki, Helsinki, Finland

**Keywords:** Cancer in the nervous system, CNS cancer

## Abstract

High-grade gliomas (HGGs) are the leading cause of brain cancer-related death. HGGs include clinically, anatomically and molecularly distinct subtypes that stratify into diffuse midline gliomas (DMGs), such as *H3K27M*-altered diffuse intrinsic pontine glioma, and hemispheric HGGs, such as IDH wild-type glioblastoma. Neuronal activity drives glioma progression through paracrine signalling^1,2^ and neuron-to-glioma synapses^3–6^. Glutamatergic AMPA receptor-dependent synapses between neurons and glioma cells have been demonstrated in paediatric^3^ and adult^4^ high-grade gliomas, and early work has suggested heterogeneous glioma GABAergic responses^7^. However, neuron-to-glioma synapses mediated by neurotransmitters other than glutamate remain understudied. Using whole-cell patch-clamp electrophysiology, in vivo optogenetics and patient-derived orthotopic xenograft models, we identified functional, tumour-promoting GABAergic neuron-to-glioma synapses mediated by GABA_A_ receptors in DMGs. GABAergic input has a depolarizing effect on DMG cells due to NKCC1 chloride transporter function and consequently elevated intracellular chloride concentration in DMG malignant cells. As membrane depolarization increases glioma proliferation^3,6^, we found that the activity of GABAergic interneurons promotes DMG proliferation in vivo. The benzodiazepine lorazepam enhances GABA-mediated signalling, increases glioma proliferation and growth, and shortens survival in DMG patient-derived orthotopic xenograft models. By contrast, only minimal depolarizing GABAergic currents were found in hemispheric HGGs and lorazepam did not influence the growth rate of hemispheric glioblastoma xenografts. Together, these findings uncover growth-promoting GABAergic synaptic communication between GABAergic neurons and *H3K27M*-altered DMG cells, underscoring a tumour subtype-specific mechanism of brain cancer neurophysiology.

## Abstract (web summary)

Functional, tumour-promoting GABAergic neuron-to-glioma synapses in diffuse midline gliomas are identified.

## Main

DMG is a lethal childhood central nervous system cancer with few therapeutic options and a median survival of only 11–13 months^8,9^. The majority of DMGs exhibit a mutation in genes encoding histone H3 (H3K27M) and occur in the brainstem, thalamus and spinal cord^10–12^; DMGs occur most commonly in the brainstem and are also known as diffuse intrinsic pontine glioma (DIPG) when in this neuroanatomical location. Multiple lines of evidence support the concept that DMG originates from oligodendroglial lineage precursor cells^13–19^. During postnatal development and adulthood, oligodendroglial precursor cells (OPCs) communicate with neurons through both paracrine factor signalling^20–22^ and through glutamatergic and GABAergic neuron-to-OPC synapses^23–27^. In contrast to the hyperpolarizing (inhibitory) effect of GABA on mature neurons, GABAergic neuron-to-OPC synapses are depolarizing due to high OPC intracellular chloride concentration, and GABAergic signalling is crucial for myelin development^24,28^. During development and in brain regions that exhibit myelin plasticity, OPC proliferation is robustly regulated by neuronal activity^20,29^. Similar to these effects on their normal cellular counterparts, glutamatergic neuronal activity drives the proliferation and growth of DMG and other high-grade^1,3,4,30,31^ and low-grade^2^ gliomas. The mechanisms by which neuronal activity promotes glioma progression include activity-regulated paracrine factor secretion^1,2,30,31^ as well as electrochemical communication through AMPA (α-amino-3-hydroxy-5-methyl-4-isoxazole propionic acid) receptor (AMPAR)-mediated neuron-to-glioma synapses^3,4^ and activity-dependent, potassium-evoked glioma currents that are evident in both paediatric and adult forms of high-grade gliomas^3,4^. Depolarizing current alone is sufficient to drive malignant glioma growth in orthotopic xenograft models^3,6^, underscoring the need for a comprehensive understanding of electrochemical mechanisms that enable glioma membrane depolarization in each molecularly distinct form of glioma. Here we tested the hypothesis that, similar to normal OPCs, depolarizing GABAergic synapses exist between GABAergic interneurons and H3K27M-altered (H3K27M^+^) DMG cells and promote tumour progression in H3K27M^+^ DMG.

## GABA_A_ receptor gene expression in DMG

**Fig. 1:**
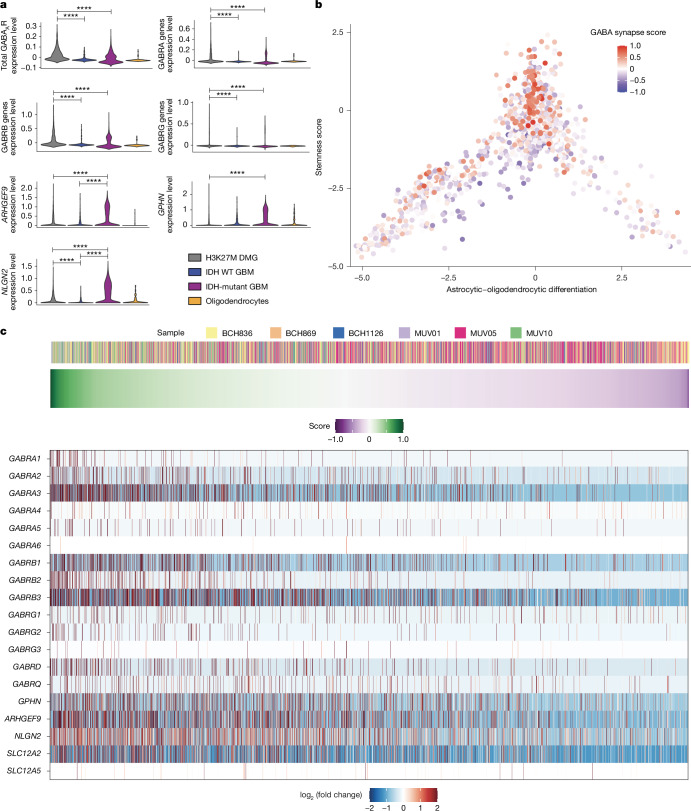
GABAergic synapse-related gene expression in glioma. **a**, Single-cell RNA-seq analysis of malignant cells from primary human biopsies of H3K27M^+^ DMG (grey; *n* = 2,259 cells, 6 study participants), IDH WT high-grade glioma (IDH WT GBM; blue; *n* = 599 cells, 3 participants) and IDH-mutant high-grade glioma (IDH-mutant GBM; purple; *n* = 5,096 cells, 10 participants) malignant cells, and tumour-associated, non-malignant oligodendrocytes (yellow; *n* = 232 cells)^15,32^, demonstrating expression of total GABA_A_R subunit genes, α-subunit genes, β-subunit genes, γ-subunit genes and postsynaptic genes specific to GABAergic synapses. Statistical analyses performed on single cells are represented with asterisks only when also significant when analysed on a per-patient basis as well as a per-cell basis (****P < 2.2 × 10^−16^ for all asterisk comparisons). Comparisons to oligodendrocytes (control cell type) are not shown. Two-tailed Wilcoxon rank-sum test. **b**, Plot of lineage score (astrocytic–oligodendrocytic differentiation) and stemness score from single-cell RNA-seq analysis of H3K27M^+^ DMG malignant single cells sorted from primary biopsies (*n* = 2,259 cells, 6 study participants). The red–blue colour overlay indicates the relative score for GABAergic synapse-related genes. **c**, GABAergic synapse-related relative gene expression in H3K27M^+^ DMG malignant cells from primary biopsies. Cells are annotated according to patient sample (top) and are sorted from left to right based on their GABA synapse-related gene expression score, as indicated by the green-to-purple gradient (middle); a heatmap of expression of individual GABAergic synapse-related genes in each DMG cell is also shown (bottom).

**Extended Data Fig. 1:**
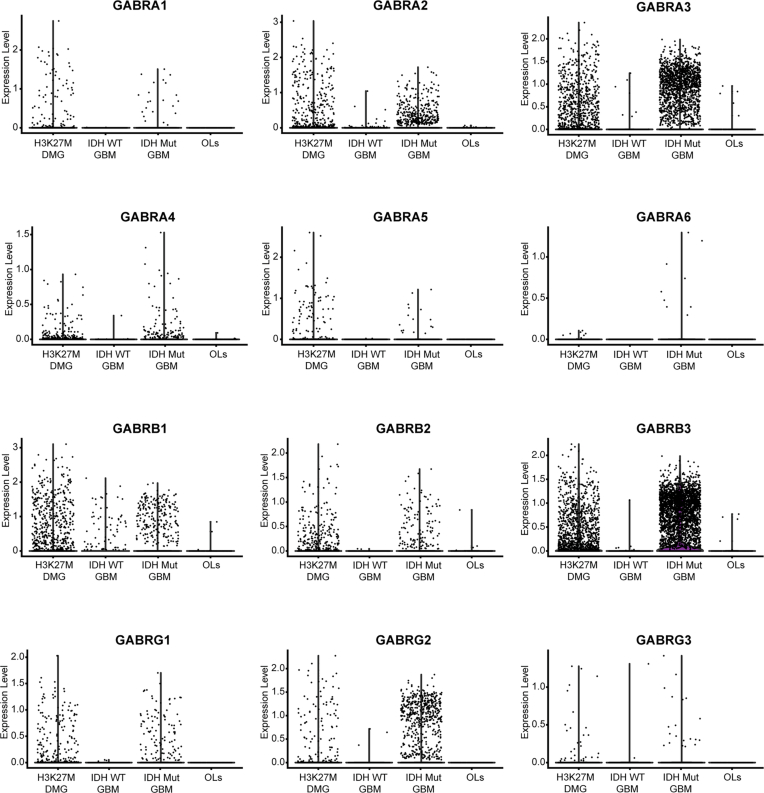
GABA_A_ subunit expression patterns in gliomas. Single cell RNAseq analysis illustrating expression of GABA_A_ subunit genes in patient-derived DMG xenografts (grey; n = 335 cells, 4 mice)^3^ and primary human biopsies of H3K27M^+^ diffuse midline glioma (DMG; grey; n = 2,259 cells, 6 study participants), IDH-wild-type (WT) high-grade glioma (GBM; blue; n = 599 cells, 3 participants), and IDH-mutant (mut) high-grade glioma (GBM; purple; n = 5,096 cells, 10 participants) malignant cells^15,32^.

**Extended Data Fig. 2:**
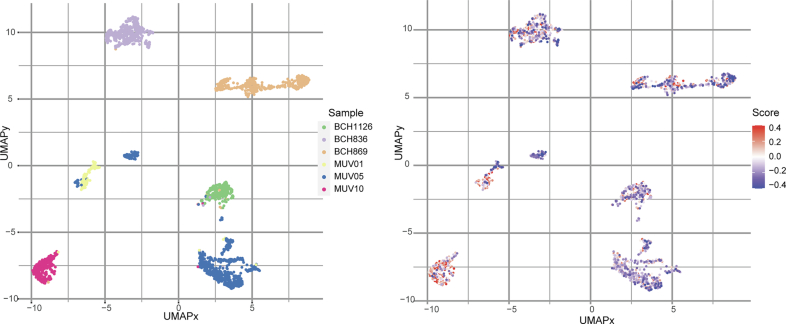
Inter- and intra-patient heterogeneity of GABAergic synapse-related gene expression. UMAP of H3K27M^+^ DMG malignant cells from primary biopsies, colored once by sample (left) and once by GABA synapse-related gene score (red to blue, right).

To determine whether genes involved in GABAergic synaptic transmission are expressed in high-grade gliomas, we analysed single-cell RNA sequencing (RNA-seq) datasets^15,32^ from the malignant cells of primary patient tumour samples of H3K27M^+^ DMG, isocitrate dehydrogenase (IDH) wild-type (WT) hemispheric high-grade glioma, IDH-mutant hemispheric high-grade glioma and tumour-associated non-malignant oligodendrocytes. H3K27M^+^ DMG cells broadly expressed GABA_A_ receptor (GABA_A_R) subunit genes, including α-subunits, β-subunits and γ-subunits, as well as *GPHN* (encoding gephyrin), *ARHGEF9* (encoding Rho guanine nucleotide exchange factor 9) and *NLGN2* (encoding neuroligin 2), which are associated with GABAergic postsynaptic compartments (Fig. 1a and Extended Data Fig. 1). These genes were expressed to a much greater extent in H3K27M^+^ DMG and IDH-mutant high-grade gliomas than in IDH WT high-grade gliomas (Fig. 1a). Gliomas consist of malignant cellular subpopulations resembling oligodendroglial and astrocytic cells; in primary patient biopsy samples of H3K27M^+^ DMG, single-cell transcriptomic analyses revealed that the GABAergic synapse-related gene expression signature is present in all malignant cellular compartments and enriched in the OPC-like cellular compartment (Fig. 1b). H3K27M^+^ DMG single cells exhibit heterogeneity in GABAergic synapse-related gene expression both between and within patient samples, with all patient tumours examined expressing GABAergic synapse-related genes to a variable extent (Fig. 1c and Extended Data Fig. 2). H3K27M^+^ DMGs express relatively high levels of GABA_A_R subunits α3, β3 and both γ-subunits, and the chloride transporter NKCC1 (Fig. 1c).

## Structural GABAergic DMG synapses

**Fig. 2:**
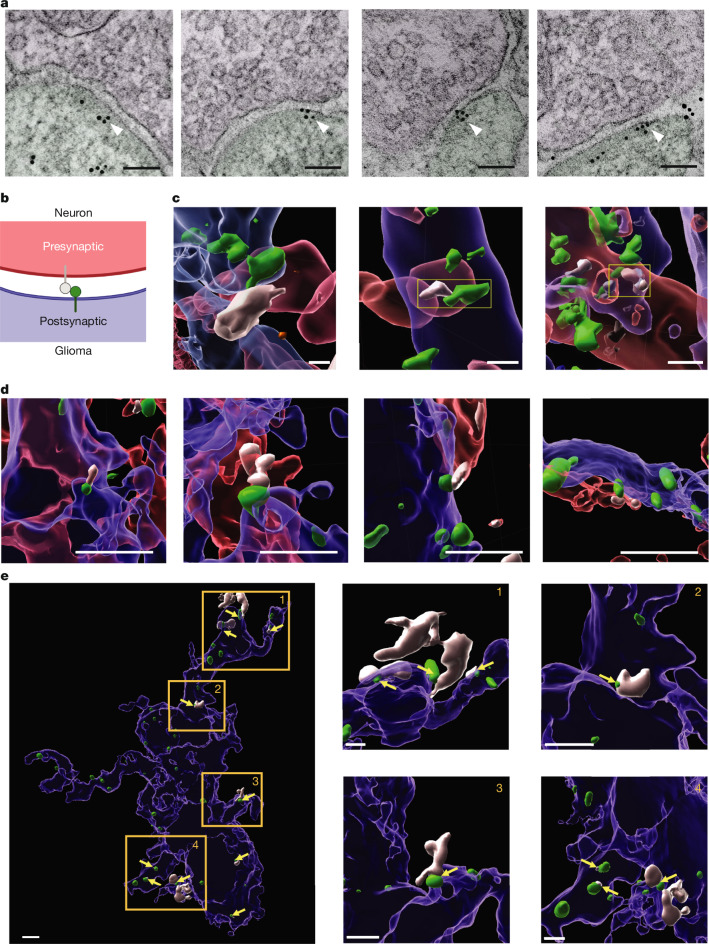
Structural GABAergic neuron-to-glioma synapses in DMG. **a**, Immuno-electron microscopy of SU-DIPG-VI cells with GFP-tagged GABRG2 xenografted into the mouse hippocampus. The white arrowheads denote immunogold labelling of GFP (glioma GABRG2). Presynaptic neurons are shaded purple, and glioma cells are shaded green. Scale bars, 100 nm. *n* = 39 synapses identified from 4 mice. **b**, Schematic of colours representing structures in GABAergic neuron-to-glioma synapses imaged in panels **c**,**d**, including neurons (red), gliomas (blue), presynaptic puncta (white) and postsynaptic puncta (green). The schematic was created using BioRender (https://biorender.com). **c**, Representative confocal micrograph 3D reconstructions of GABAergic neuron-to-glioma synaptic puncta in neuron–glioma co-cultures. Patient-derived DMG cells (SU-DIPG-XIII-FL; nestin in blue) expressing GABRG2–GFP (postsynaptic marker in green) colocalize with the presynaptic marker synapsin (white) on rat hippocampal neurons (neurofilament in red). Scale bars, 2 µm. **d**, Representative confocal micrograph 3D reconstructions of GABAergic neuron-to-glioma synaptic puncta in patient-derived xenografts. Postsynaptic gephyrin (green) on GFP-expressing patient-derived DMG cells (SU-DIPG-XIII-FL; GFP in blue) colocalizes with the presynaptic marker VGAT (white) on neurons (DLX–mCherry in red) in the mouse hippocampus. Scale bars, 2 µm. **e**, Representative confocal micrograph 3D reconstruction of a glioma cell (blue) with several gephyrin puncta (green) colocalized with presynaptic VGAT puncta (white). Colocalized puncta are indicated with yellow arrows and some examples are highlighted in yellow boxes. Scale bars, 2 µm.

**Extended Data Fig. 3:**
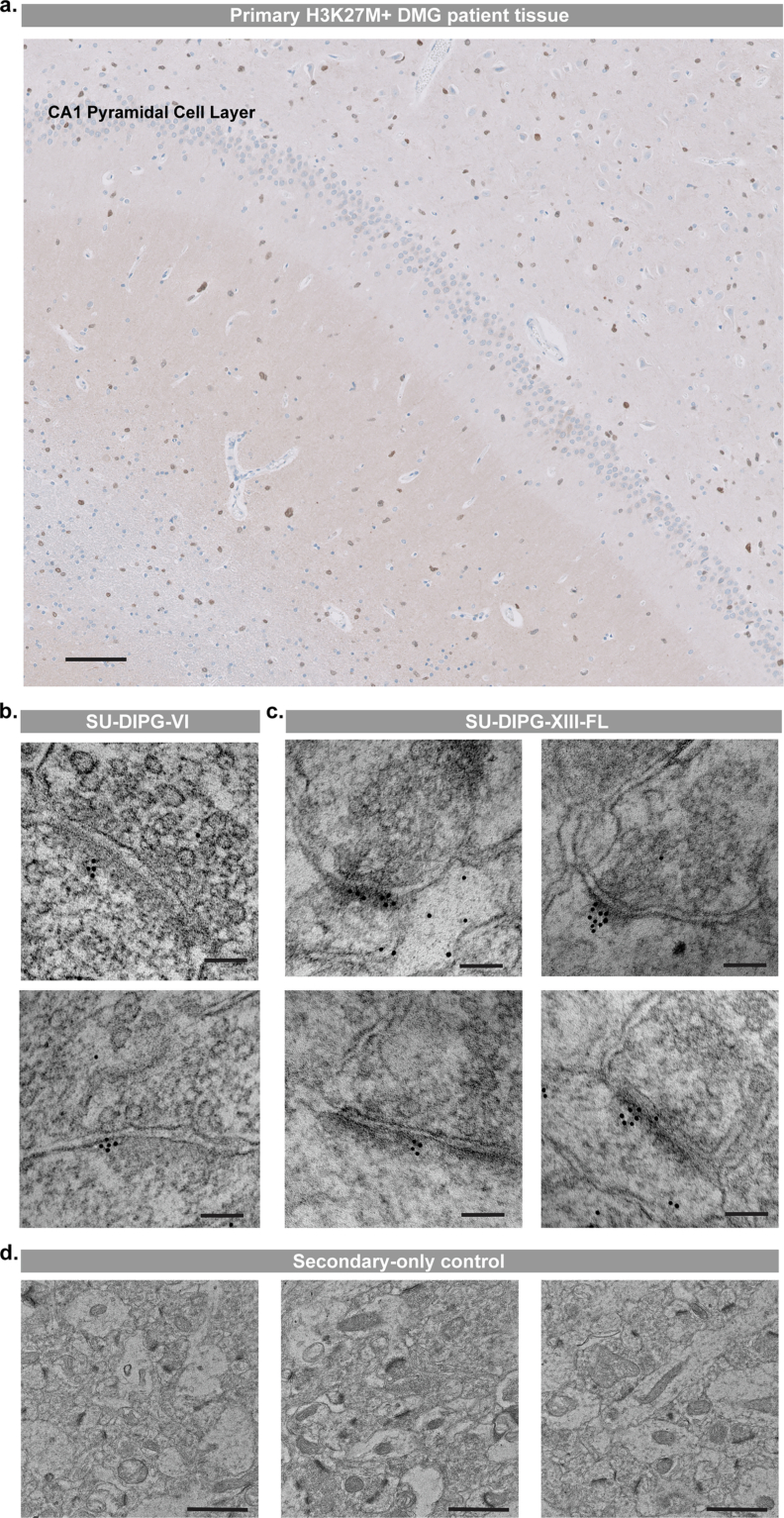
DMG infiltration of human hippocampus and immuno-electron micrographs of GABAergic neuron-to-glioma synapses in hippocampal DMG xenografts. **a**. DMG infiltration of human hippocampus. 10x magnification light microscopy image of primary patient tissue with H3K27M^+^ DMG cells (brown) in the CA1 region of the hippocampus. Hematoxylin counterstain. DMG infiltration of hippocampus is not uncommon, as this case of a 14-year-old female with H3K27M-mutated DMG, also called diffuse intrinsic pontine glioma (DIPG), illustrates. Patient sample taken at time of autopsy. Scale bar, 100 µm. **b-c**. Immuno-electron microscopy of SU-DIPG-VI (n = 39 synapses identified from 4 mice, **b**) and SU-DIPGXIII-FL (n = 97 synapses identified from 3 mice, **c**) cells with GFP-tagged GABRG2 xenografted into mouse hippocampus. Six examples of GABAergic neuron-to-glioma synapses are shown here, in addition to those shown in Fig. 2a. Scale bar, 100 nm. **d**. Secondary-only control for immuno-electron microscopy of SU-DIPGXIII-FL with GFP-tagged GABRG2 xenografted into mouse hippocampus to demonstrate low background immuno-gold labelling. Scale bar, 1000 nm. n = 7 biological replicates.

**Extended Data Fig. 4:**
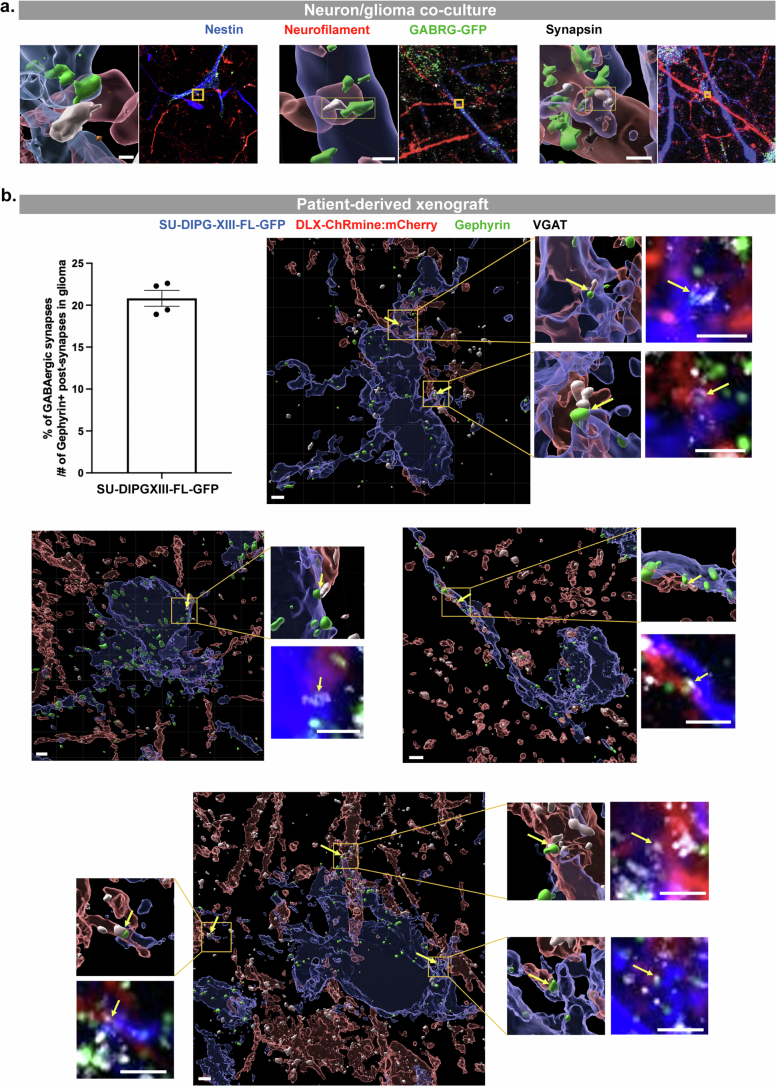
GABAergic neuron-to-glioma synaptic puncta imaging and 3D reconstruction. **a**. 3-dimensional (3D) reconstruction shown in Fig. 2c and associated zoomed-out confocal images of GABAergic neuron-to-glioma synaptic puncta in neuron-glioma co-cultures. H3K27M^+^ DMG cells (SU-DIPGXIII-FL, nestin, blue) expressing GARBRG2-GFP (green, post-synaptic) co-localizes with synapsin (white, pre-synaptic) on rat hippocampal neurons (neurofilament, red). Scale bars, 2 µm. **b**. Zoomed-out 3-dimensional (3D) reconstructions and zoomed-in 3D reconstructions (as shown in Fig. 2d,e) with associated confocal images of GABAergic neuron-to-glioma synaptic puncta in patient-derived DMG xenografts in the mouse hippocampus. Gephyrin (green) on GFP-expressing DMG cells (SU-DIPGXIII-FL, GFP,blue) co-localizes with VGAT (white) on neurons (DLX-mCherry, red). Scale bars, 2 µm. Top left, quantification of co-localized synaptic puncta as a percent of total gephyrin puncta on glioma cells (n = 4 mice). Select colocalized puncta are indicated with yellow arrows, and some examples are highlighted in yellow boxes. Please note that several 3D-reconstructed images from Fig. 2 are shown again here to provide additional spatial and contextual information. All data are mean ± s.e.m. Source Data

We sought to visualize GABAergic synapses between GABAergic neurons and DMG tumour cells. First, we expressed a construct encoding the GABA_A_ subunit *GABRG2* tagged with GFP in patient-derived DMG tumour cells and subsequently xenografted these GABA_A_R subunit-tagged tumour cells to the mouse hippocampus, a region to which DMG frequently spreads (Extended Data Fig. 3a), allowing engraftment for 8 weeks. Immuno-electron microscopy was then performed against GFP to unambiguously identify neuron-to-glioma GABAergic synapses containing the GFP-tagged GABA_A_R subunits (Fig. 2a and Extended Data Fig. 3b). In a second set of experiments, patient-derived DMG cells expressing *GABRG2–GFP* were co-cultured with mixed GABAergic and glutamatergic mouse neurons^3,6^. Immunocytochemistry for neuronal (neurofilament) and glioma (nestin) markers, together with a presynaptic marker (synapsin 1) and GABRG2–GFP revealed colocalization of neuronal presynaptic puncta with glioma postsynaptic GABRG2–GFP puncta by confocal microscopy (Fig. 2b,c, Extended Data Fig. 4a and Supplementary Video 1). Next, we used a third strategy to visualize neuron-to-glioma GABAergic synapses. In mice expressing mCherry in GABAergic interneurons under the *DLX* promoter, patient-derived DMG cells expressing cytoplasmic GFP were xenografted to the mouse hippocampus in vivo and allowed to engraft for 8 weeks. Immunohistochemistry for GABAergic interneurons (mCherry) and DMG tumour cells (GFP) together with a GABAergic presynaptic marker (VGAT) and GABAergic postsynaptic marker (gephyrin, a nearly universal component of GABAergic postsynaptic structures) revealed colocalization of interneuronal presynaptic puncta (VGAT) with glioma postsynaptic gephyrin puncta by confocal microscopy (Fig. 2d,e and Extended Data Fig. 4b). Quantifying synaptic, colocalized gephyrin puncta and non-colocalized, extrasynaptic gephyrin puncta on DMG glioma cells following confocal microscopy and 3D reconstruction, we found that approximately 20% of glioma cell gephyrin puncta are colocalized with presynaptic VGAT (Extended Data Fig. 4b). Together, these complementary experimental strategies demonstrate structural GABAergic synapses between GABAergic interneurons and patient-derived DMG cells.

## Functional GABAergic DMG synapses

**Fig. 3:**
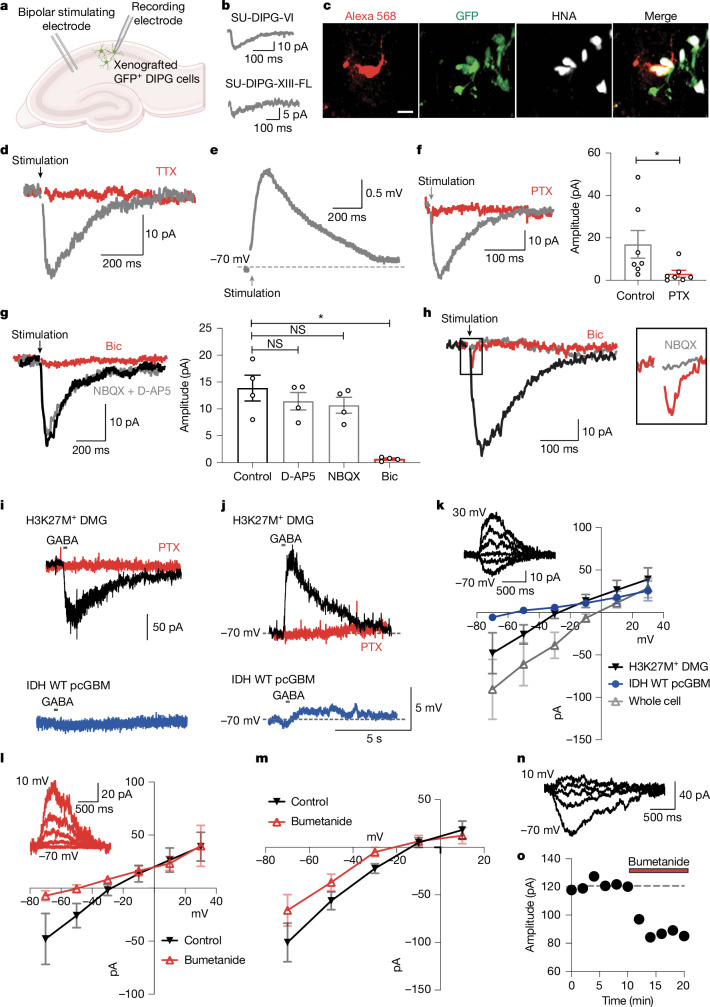
Depolarizing GABAergic neuron-to-glioma synapses in DMG. **a**, Schematic depicting the electrophysiological recording of DMG cells xenografted into hippocampal CA1 in response to local stimulation. The schematic was created using BioRender (https://biorender.com). **b**, Representative traces (grey) of currents elicited by electrical stimulation in the presence of NBQX in two patient-derived DMG xenografts (SU-DIPG-VI and SU-DIPG-XIII-FL). **c**, Xenografted DMG (SU-DIPG-VI) cell dye filled (Alexa 568; red) during recording and co-labelled with GFP (green) and human nuclear antigen (HNA; white) post-recording. Scale bar, 10 µm. *n* = 5 biological replicates. **d**, Representative voltage-clamp trace of a tetrodotoxin (TTX; red)-sensitive DMG (SU-DIPG-VI) cell current in the presence of NBQX (grey). **e**, Representative trace of stimulation-evoked voltage change in a DMG (SU-DIPG-VI) cell in the presence of NBQX (grey) using current clamp at −70 mV. **f**, Representative voltage-clamp trace of the picrotoxin (PTX; red)-sensitive GABAergic postsynaptic current (PSC) in a DMG (SU-DIPG-VI) cell in the presence of NBQX (grey; left), and quantification of the current amplitude (right; *n* = 7 cells from 5 mice; *P* = 0.0341). Paired two-tailed Student’s *t*-test. **g**, Representative voltage-clamp trace of GABAergic PSC in a DMG (SU-DIPG-VI) cell with NBQX + D-AP5 (grey) and bicuculline (Bic; red; left), or no inhibitors (black trace), and quantification of the current amplitude (right; *n* = 5 cells from 3 mice; *P* = 0.1787 (control versus D-AP5), *P* = 0.1093 (control versus NBQX) and *P* = 0.0287 (control versus Bic)). Repeated measures one-way ANOVA with Dunnett’s post-hoc test. **h**, Representative voltage-clamp trace of a DMG (SU-DIPG-VI) cell response to stimulation demonstrating Bic-sensitive GABAergic PSC (left) and NBQX-sensitive glutamatergic PSC in the same DMG cell (right). Red trace, in the presence of Bic; grey trace, in the presence of NBQX; black trace, no inhibitors. **i**,**j**, Representative traces of perforated patch recordings from xenografted patient-derived DMG (SU-DIPG-VI) and hemispheric high-grade glioma (SU-pcGBM-2) cells in voltage clamp (**i**) and current clamp (**j**) in response to GABA. Black traces, H3K27M^+^ DMG with no inhibitors; red traces, H3K27M^+^ DMG in the presence of PTX; blue traces, hemispheric high-grade glioma. **k**, GABA current–voltage relationship of perforated patch recordings in DMG cells (SU-DIPG-VI; black; *n* = 6 cells from 5 mice) and H3/IDH WT hemispheric high-grade glioma cells (SU-pcGBM-2; blue; *n* = 6 cells from 5 mice), and whole-cell patch with high internal Cl^−^ concentration in DMG cells (SU-DIPG-VI; grey; *n* = 4 cells from 4 mice). Representative traces of a DMG cell response to GABA at membrane potentials from −70 mV to +30 mV are also shown (inset). **l**, GABA current–voltage relationship of perforated patch recordings in DMG (SU-DIPG-VI) cells with no inhibitor (black) or in the presence of 100 μM bumetanide (red; *n* = 5 cells from 3 mice). Representative traces of membrane potentials from −70 mV to +10 mV with bumetanide are also shown (inset). **m**, GABA current–voltage relationship in DMG (SU-DIPG-VI) cells in whole-cell patch clamp with low internal Cl^−^ concentration (negative Cl^−^ load) in the presence (red) or absence (black) of 10 µM bumetanide (*n* = 5 cells from 3 mice). **n**, Representative voltage-clamp trace of the GABA current in DMG (SU-DIPG-VI) cells at membrane potentials from −70 mV to +10 mV under negative Cl^−^ load conditions. **o**, Representative time course of bumetanide (10 µM) effect on GABA current in DMG (SU-DIPG-VI) cells at −70 mV under negative Cl^−^ load conditions. Dashed line indicates baseline amplitude before addition of bumetanide. All data are mean ± s.e.m. NS, not significant, *P < 0.05. Source Data

To determine whether the observed structural GABAergic neuron-to-glioma synapses are functional, we performed patch-clamp electrophysiological recordings from xenografted H3K27M^+^ DMG cells. GFP-expressing glioma cells were xenografted into the CA1 region of the mouse hippocampus—a well-defined circuit in which neuron-to-glioma synapses have been previously studied^3^—and allowed to engraft for at least 8 weeks. DMG cell responses to low-intensity electrical stimulation of local neurons (a distance of 100–200 μm from the patched DMG cell) were recorded using whole-cell patch-clamp electrophysiology with a high-chloride internal solution in the patch pipettes to facilitate the detection and quantification of GABA_A_R-mediated responses in acute hippocampal slices (Fig. 3a). Under voltage clamp conditions in the presence of the AMPAR antagonist NBQX (2,3-dihydroxy-6-nitro-7-sulfamoyl-benzo[f]quinoxaline) to block AMPAR-mediated glutamatergic neuron-to-glioma synapses^3,4,6,33^, local stimulation led to an inward current consistent with a GABAergic synaptic response observed in approximately 40% (31 of 78 and 3 of 7) of DMG cells in two distinct patient-derived DMG xenograft models (Fig. 3b). These responses had a rise time of 18.5 ± 2.6 ms and a decay time of 106.6 ± 6.5 ms with a response latency of 4.8 ± 0.7 ms, as measured by the time from the end of the 0.5-ms stimulus to the beginning of the response. The slow decay time is consistent with currents through GABA_A_Rs containing the α3 subunit^34,35^, including α3β3γ2 GABA_A_Rs^36^, concordant with single-cell RNA-seq data showing that these are the most highly expressed GABA_A_R subunits in DMGs (Extended Data Fig. 1). Tonic and extrasynaptic GABA signalling may also contribute to the slow decay time. GABAergic currents consistent with synaptic transmission could be reliably evoked, and, as expected, failures were not observed using this multi-axon stimulation paradigm. Cells were filled with Alexa Fluor 568 dye in the whole-cell patch solution during electrophysiological recordings to subsequently confirm that the cells recorded from were glioma cells (Fig. 3c).

These GABAergic currents were elicited by neuronal action potentials, as the voltage-gated sodium channel blocker tetrodotoxin application prevented the glioma currents (Fig. 3d). As seen in current clamp experiments, these inwardly directed glioma currents were strongly depolarizing due to the high concentration of Cl^−^ in the internal solution (Fig. 3e). These currents were blocked by the GABA_A_R inhibitors picrotoxin and bicuculline, but not by the AMPAR inhibitor NBQX and the NMDAR inhibitor D-AP5 (Fig. 3f,g). Blocking the GABAergic response in the absence of glutamate receptor antagonists revealed an NBQX-sensitive glutamatergic synaptic current in a subpopulation of DMG cells (Fig. 3h), indicating that both GABAergic and glutamatergic neurons can synapse onto the same DMG cell. It should be noted that the amplitudes of the GABAergic and glutamatergic currents are not comparable to each other given the high chloride concentration in the patch pipette. Next, we examined the response of DMG cells to GABA while maintaining their native chloride concentration.

## GABA depolarizes DMG cells

GABA_A_R activation can result in the flux of negatively charged chloride ions either out of or into a cell, thereby either depolarizing or hyperpolarizing the membrane potential, depending on the electrochemical gradient of Cl^−^ ions across the cell membrane. In mature central nervous system neurons, the intracellular Cl^−^ concentration is actively maintained at a low level by the K–Cl cotransporter KCC2, leading to an influx of Cl^−^ in response to activation of GABA_A_Rs and thus hyperpolarization^37,38^. By contrast, OPCs and several other types of non-neuronal cells in the brain express high levels of the Na–K–2Cl cotransporter NKCC1 (ref. ^39^), leading to a high intracellular Cl^−^ concentration, and consequently to an efflux of Cl^−^ upon activation of GABA_A_Rs; thus GABAergic neuron-to-OPC synapses cause depolarization^24,28^. To determine the effect of GABA on the membrane potential of H3K27M^+^ DMG cells, we performed perforated patch recordings of xenografted DMG cells using gramicidin-A, which does not perturb the intracellular concentration of Cl^−^. Local application of GABA induced a robust inward current in voltage clamp and corresponding depolarization in current clamp of DMG cells (Fig. 3i,j). Consistent with the low GABA_A_ receptor gene expression profile of H3/IDH WT glioblastoma (GBM; Fig. 1a), local GABA application on H3/IDH WT paediatric hemispheric GBM (paediatric cortical GBM (pcGBM)) xenografted slices evoked a negligible current in comparison (Fig. 3i,j). The depolarizing nature of these GABAergic currents in DMG indicate a high intracellular Cl^−^ concentration in DMG cells that is maintained by active Cl^−^ uptake. Both the current and the voltage responses were fully blocked by the GABA_A_R antagonist picrotoxin, which rules out a contribution of GABA_B_Rs (Fig. 3i,j). These responses to exogenous GABA mediated by GABA_A_Rs may include both synaptic and extrasynaptic currents.

**Extended Data Fig. 5:**
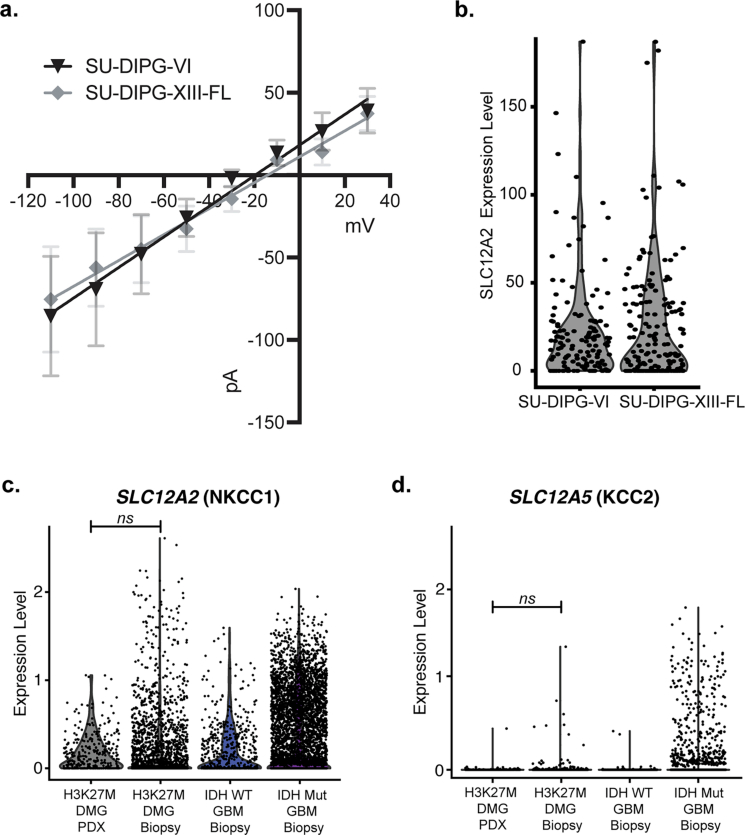
Cl^−^ transporter expression in patient-derived xenografts of H3K27M + DMG. **a**. Current-voltage relationship of GABA current in two patient-derived DMG xenograft models recorded with perforated patch. Reversal potential of GABA was −25.0 ± 3.7 mV in SU-DIPGVI cells (n = 6 cells from 5 mice), and −20.7 ± 4.9 mV in SU-DIPGXIII-FL cells (n = 5 cells from 3 mice). **b**. Single cell RNAseq analysis of SLC12A2 (NKCC1) in two DMG patient-derived xenografts (PDX). **c**,**d**. Single cell RNAseq analysis illustrating expression of *SLC12A2* (NKCC1; **c**) and *SLC12A5* (KCC2; **d**) in patient-derived DMG xenografts (grey; n = 335 cells, 4 mice)^3^ and primary human biopsies of H3K27M^+^ diffuse midline glioma (grey; n = 2,259 cells, 6 study participants), IDH-wild-type high-grade glioma (IDH WT GBM; blue; n = 599 cells, 3 participants), and IDH-mutant high-grade glioma (IDH-mut GBM; purple; n = 5,096 cells, 10 participants) malignant cells^15,32^. Two-tailed Wilcoxon Rank Sum test. All data are mean ± s.e.m. *ns*, not significant. Source Data

The intracellular chloride concentration of a cell can be inferred from the reversal potential of GABA_A_ currents (*E*_GABA_). To determine *E*_GABA_ in H3K27M^+^ DMG and H3/IDH WT pcGBM xenografts, we recorded currents evoked by local GABA application at varying holding potentials and constructed current–voltage relationship plots (Fig. 3k). The currents in response to GABA were plotted against the holding potentials, and the reversal potential for GABA (*E*_GABA_; the membrane voltage at which the net current flow through the GABA_A_ channel is zero) was found to be −25.0 ± 3.7 mV and −20.7 ± 4.9 mV for H3K27M^+^ DMG cells from two different patient-derived models (SU-DIPG-VI and SU-DIPG-XIII-FL, respectively) and −61.3 ± 7.9 mV for H3/IDH WT hemispheric high-grade glioma (patient-derived model SU-pcGBM-2; Fig. 3k and Extended Data Fig. 5a). Using these reversal potentials and the Nernst equation to calculate the intracellular Cl^−^ concentration of each glioma type, we found that the intracellular Cl^−^ concentration of H3K27M^+^ DMG cells is approximately 51 mM in SU-DIPG-VI and approximately 60 mM in SU-DIPG-XIII-FL, and in H3/IDH WT hemispheric high-grade glioma the intracellular Cl^−^ concentration is approximately 13 mM (SU-pcGBM-2). During whole-cell recordings with high Cl^−^ internal patch pipette solution that sets the intracellular Cl^−^ concentration close to that of the extracellular solution, the reversal potential was close to zero (−1.4 ± 5.2 mV) in H3K27M^+^ DMG cells, illustrating the critical role of chloride concentration gradients (Fig. 3k).

## NKCC1 elevates Cl^−^ in DMG cells

As discussed above, cation–chloride cotransporters, particularly the Na–K–Cl cotransporter NKCC1 and the K–Cl cotransporter KCC2, have an important role in setting intracellular Cl^−^ concentration in neurons. *SLC12A2*, the gene that encodes NKCC1, which transports Cl^−^ into the cell, is expressed in H3K27M^+^ DMG primary patient biopsies and patient-derived xenografts (Fig. 1c and Extended Data Fig. 5b,c). By contrast, *SLC12A5*, the gene that encodes KCC2, which extrudes Cl^−^, is expressed at very low levels in DMGs (Fig. 1c and Extended Data Fig. 5d). Hypothesizing that the NKCC1 chloride transporter is responsible for the high intracellular Cl^−^ concentration and consequent depolarizing effect of GABA in DMG cells, we used the perforated patch method to record the response to local GABA application in the presence of bumetanide, a potent and selective NKCC1 inhibitor in brain tissue. After incubation with bumetanide, *E*_GABA_ was shifted from −25.0 ± 3.7 mV to −57.2 ± 7.4 mV in H3K27M^+^ DMG cells (Fig. 3l), a value similar to that found in H3/IDH WT pcGBM cells (Fig. 3k). A standard approach to study the efficacy of ion transport is to impose an external load on a given transporter by manipulating the relevant ion gradient^40^. Therefore, we carried out whole-cell clamp experiments using pipettes with a low Cl^−^ concentration (10 mM) to impose a ‘negative Cl^−^ load’ on NKCC1 in DMG cells, requiring NKCC1 to transport Cl^−^ more effectively into the DMG cell. Blocking NKCC1 with bumetanide in this experimental paradigm again resulted in a shift in *E*_GABA_ from −5.2 ± 4.3 mV to −9.3 ± 2.8 mV within the same cell before and after bumetanide perfusion, respectively (Fig. 3m–o). Together, these results indicate that NKCC1 function is critical for the depolarizing effect of GABA in DMG cells.

## GABAergic neurons increase DMG growth

**Fig. 4:**
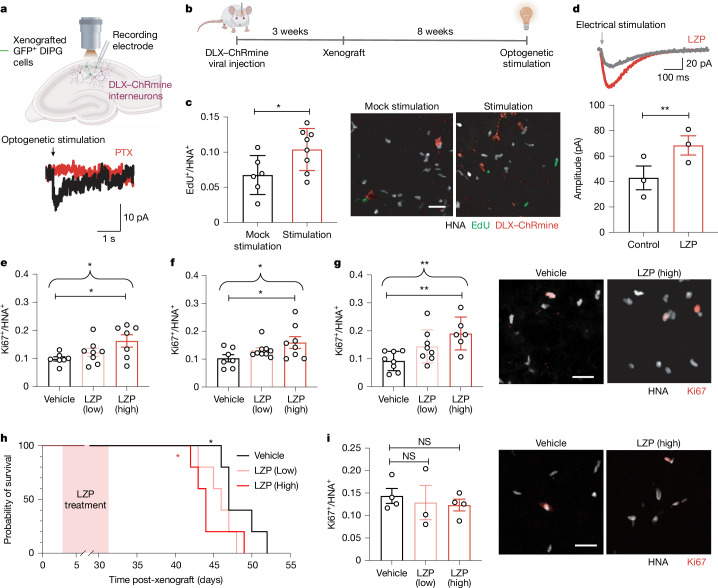
GABAergic signalling drives DMG proliferation. **a**, Electrophysiological recording of DMG cells xenografted into hippocampal CA1 in response to optogenetic stimulation of interneurons (top), and PTX-sensitive glioma current in response to optogenetic stimulation of GABAergic interneurons (bottom). The diagram was created using BioRender (https://biorender.com). **b**, In vivo optogenetic stimulation of DLX–ChRmine interneurons near xenografted DMG cells in the CA1 region of the hippocampus. The illustrations were created using BioRender (https://biorender.com). **c**, Proliferation index (EdU^+^/HNA^+^ cells) after optogenetic stimulation or mock stimulation (*n* = 6 mice; stimulation, *n* = 8 mice, *P* = 0.0075; two-tailed Student’s *t*-test; left), and representative images of DLX–ChRmine interneurons (red) near xenografted DMG cells expressing EdU (green) and HNA (white; right). Scale bar, 25 µm. **d**, Representative trace of GABAergic PSC in DMG elicited by electrical stimulation in the presence of NBQX before and after perfusion of 10 μM lorazepam (LZP; top), and quantification of current amplitude (*n* = 3 cells from 3 mice; *P* = 0.0386, two-tailed paired Student’s *t*-test; right). **e**–**g**, H3K27M^+^ diffuse midline glioma: dose-dependent (2 mg kg^−1^ (low) and 8 mg kg^−1^ (high)) effect of LZP treatment in mice with patient-derived DMG xenografts, SU-DIPG-XIII-FL (*n* = 7 mice (vehicle), *n* = 8 mice (low) and *n* = 7 mice (high); *P* = 0.0374 (**e**)), SU-DIPG-50 (*n* = 7 mice (vehicle), *n* = 9 mice (low) and *n* = 8 mice (high); *P* = 0.0573 (**f**)) and SU-DIPG-VI (*n* = 8 mice (vehicle), *n* = 8 mice (low) and *n* = 6 mice (high); *P* = 0.0075 (**g**); one-way ANOVA). The straight brackets denote Dunnett’s multiple comparisons test between two groups (vehicle versus high: *P* = 0.0228 (**e**), *P* = 0.033 (**f**) and *P* = 0.0041 (**g**)). The curved brackets denote a post-test for linear contrast among all groups (*P* = 0.0124 (**e**), *P* = 0.0181 (**f**) and *P* = 0.002 (**g**)). Representative images of xenografted SU-DIPG-VI cells in the pons expressing Ki67 (red) and HNA (white) are also shown (**g**, right). Scale bar, 25 µm. **h**, Kaplan–Meier survival curves of mice xenografted with patient-derived DMG cells (SU-DIPG-XIII-pons) and treated with LZP or vehicle (*n* = 5 mice per group), demonstrating a significant reduction in survival in mice treated with a high dose of LZP (red asterisk; *P* = 0.0495, Mantel–Cox test), and a dose-dependent reduction in survival (black asterisk; *P* = 0.0320, log-rank test for trend). **i**, Hemispheric high-grade glioma: LZP treatment in mice with patient-derived pcGBM (SU-pcGBM2) xenografts (*n* = 4 mice (vehicle), *n* = 3 mice (low) and *n* = 4 mice (high); left), and representative images of xenografted SU-pcGBM2 cells expressing Ki67 (red) and HNA (white; right). Scale bar, 25 µm. One-way ANOVA. All data are mean ± s.e.m. *P < 0.05 and **P < 0.01. Source Data

**Extended Data Fig. 6:**
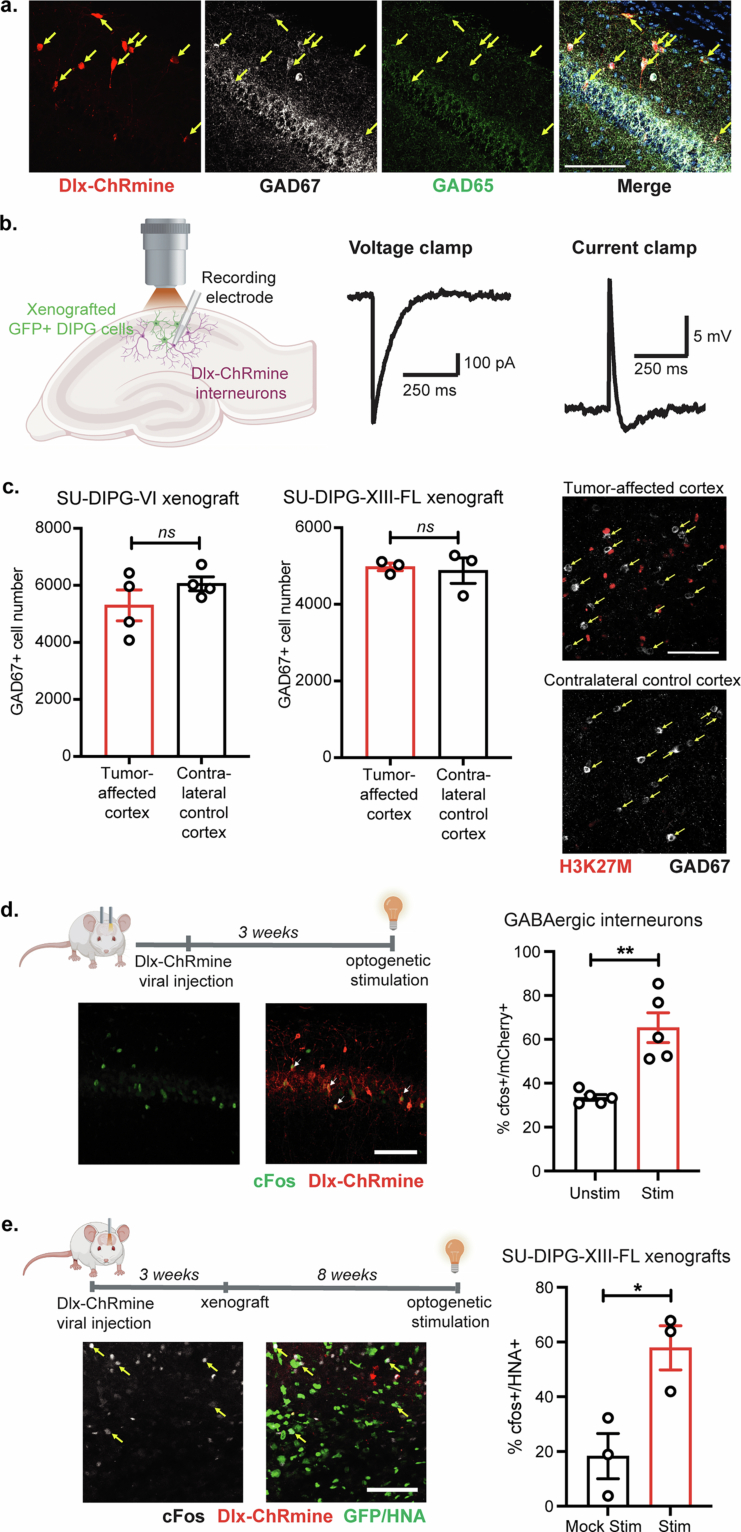
Optogenetic stimulation of GABAergic interneurons expressing DLX-ChRmine. **a**. Immunostaining and representative confocal micrographs of DLX-ChRmine (red) expression colocalized with GAD65 (green) and/or GAD67 (white). Arrows indicate co-labelled interneurons. Scale bar, 100 µm. **b**. Inward current and corresponding depolarization of GABAergic interneurons expressing DLX-ChRmine in response to optogenetic stimulation were recorded using patch clamp electrophysiology. **c**. Unbiased stereological quantification of GAD67^+^ interneurons in tumor-affected frontal cortex and contralateral control frontal cortex of mice bearing patient-derived DMG xenografts, SU-DIPGVI (n = 4 mice, p = 0.1147) and SU-DIPGXIII-FL (n = 3 mice, p = 0.829). Paired two-tailed student’s t-test. Right, representative confocal images of GAD67^+^ interneurons (white, arrows) near H3K37M^+^ DMG cells (red). Scale bar, 100 µm. **d**. Optogenetic stimulation of interneurons expressing DLX-ChRmine (red) induces neuronal activity, indicated by cfos expression (green). Arrows indicate co-labeled interneurons. Scale bar, 100 µm. Above, experimental timeline. Right, quantification of cfos expression in interneurons 90 min after optogenetic stimulation in the stimulated hemisphere (stim) indicates greater numbers of cfos-labeled interneurons than in the unstimulated hemisphere (unstim); n = 5 mice per group; p = 0.0078. Paired two-tailed student’s t-test. **e**. Optogenetic stimulation of interneurons expressing DLX-ChRmine (red) in the microenvironment of DMG xenografts increases cfos expression (white) in GFP^+^/HNA^+^ glioma cells (green). Arrows indicate co-labeled DMG cells. Scale bar, 100 µm. Above, experimental timeline. Right, quantification of cfos expression in glioma 24 h after optogenetic stimulation of interneurons in the stimulated group (stim) indicates greater numbers of cfos-labeled DMG cells than in the mock stimulated group (mock stim); n = 3 mice per group; p = 0.0264. Two-tailed student’s t-test. All data are mean ± s.e.m. *P < 0.05, **P < 0.01. The diagrams in panels **b**,**d**,**e** were created using BioRender (https://biorender.com). Source Data

Past work has demonstrated that glutamatergic neuronal activity promotes glioma progression^1–5^, and that depolarization of glioma cells has a central role in these effects of neuronal activity on glioma proliferation^3^. As GABA has a depolarizing effect on DMG cells as described above, we sought to determine whether GABAergic interneurons drive DMG proliferation through depolarizing GABAergic synaptic input. We first used whole-cell patch-clamp electrophysiology to confirm that we could perform optogenetic targeting of GABAergic neuron-to-glioma synapses. We genetically expressed ChRmine, a red-shifted channelrhodopsin^41^, in DLX-expressing GABAergic interneurons in the CA1 region of the hippocampus and confirmed their identity with immunostaining (Extended Data Fig. 6a). We recorded the electrophysiological response of xenografted glioma cells to 5-ms optogenetic stimulation of DLX–ChRmine neurons with blue (595 nm) light (Fig. 4a). Picrotoxin-sensitive GABAergic postsynaptic currents in DMG cells were consistently observed in response to optogenetic interneuron stimulation (Fig. 4a). Whole-cell patch-clamp recordings of DLX–ChRmine-expressing interneurons confirmed that optogenetic stimulation evoked depolarization (Extended Data Fig. 6b).

In IDH WT GBM, GABAergic interneurons are selectively depleted in the peritumoural cortex^42,43^. Given the differing functional^44^ and electrophysiological (Fig. 3i–k) effects of GABAergic signalling on IDH WT GBM compared with DMG, we hypothesized that GABAergic neuron loss may differ as well in the DMG-affected brain. We used unbiased stereology to quantify the number of GABAergic (GAD67^+^) interneurons in the DMG-infiltrated frontal cortex compared with the number of GABAergic (GAD67^+^) interneurons in the contralateral frontal cortex. In contrast to the marked interneuron loss observed in the GBM-infiltrated cortex^42^, we found little to no decrease in the total number of interneurons in the DMG-affected cortex compared with the non-infiltrated contralateral frontal cortex of the same mice in two distinct patient-derived xenograft models of DMG (Extended Data Fig. 6c). Together, these data indicate relative preservation of interneurons in this glioma type and further highlight the differences between GBM and DMG GABAergic biology.

Given this relatively intact population of GABAergic interneurons in the DMG microenvironment, we next sought to test the effect of interneuron activity and GABAergic synaptic input into DMG cells in vivo. DLX–ChRmine was expressed in hippocampal interneurons via adeno-associated virus vector injection to the CA1 region, and successful in vivo optogenetic stimulation of interneuron activity was confirmed by expression of the immediate early gene *Fos* (Extended Data Fig. 6d). Eleven weeks after injection of the DLX–ChRmine vector into the hippocampus, and 8 weeks after xenografting patient-derived H3K27M^+^ DMG cells to the same area, interneurons in the CA1 region of the hippocampus were optogenetically stimulated (595-nm light, 40 Hz, 30 s on/90 s off over 30 min) in awake, behaving mice (Fig. 4b). Control mice were identically manipulated, but light was not delivered during mock optogenetic stimulation. The thymidine analogue EdU was administered systemically to mice at the time of optogenetic or mock stimulation to label proliferating cells, and glioma cell proliferation was analysed 24 h later. In vivo optogenetic stimulation of GABAergic interneurons promoted proliferation of xenografted DMG cells (Fig. 4c) and also increased glioma Fos expression (Extended Data Fig. 6e).

## Benzodiazepines accelerate DMG growth

**Extended Data Fig. 7:**
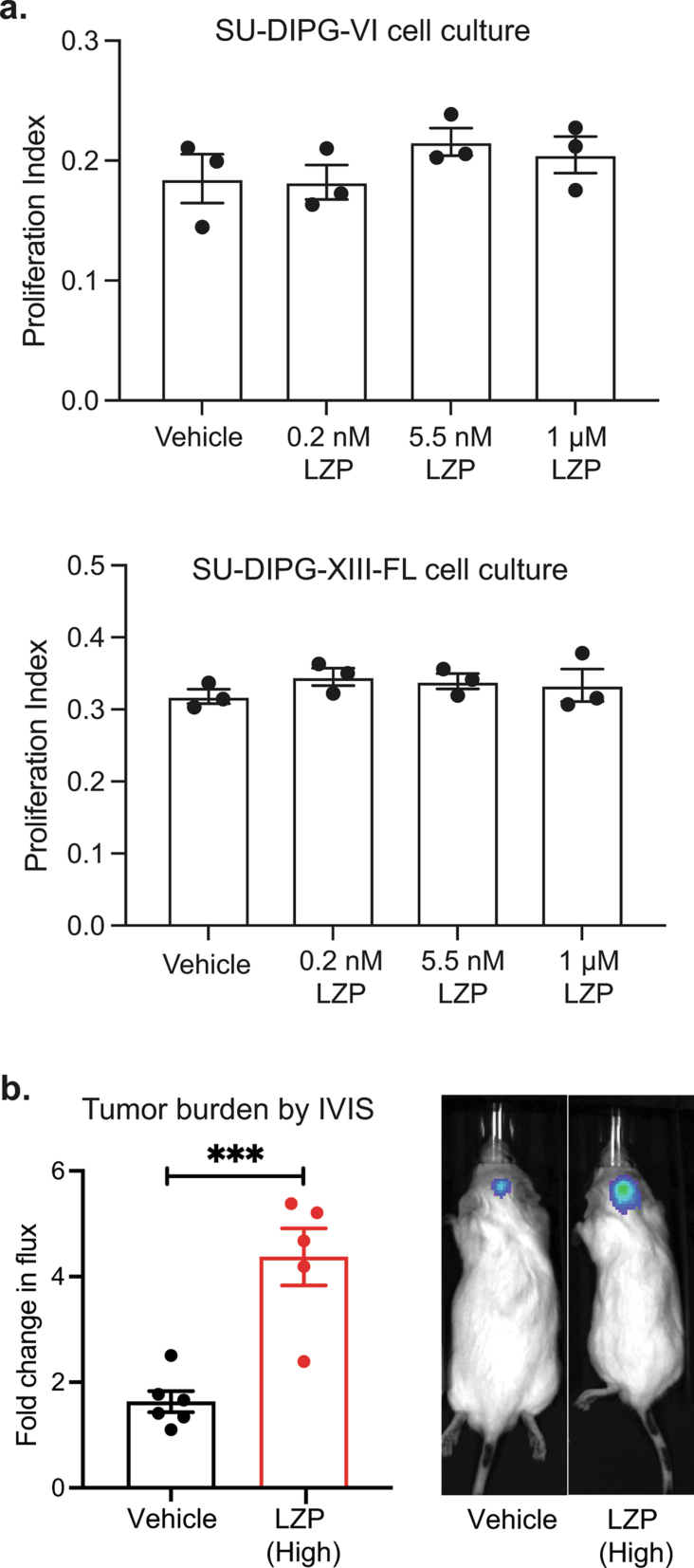
Lorazepam promotes tumor growth in vivo but has no effect on proliferation of patient-derived DMG cells in monoculture. **a**. LZP treatment of patient-derived DMG cultures SU-DIPGVI and SU-DIPGXIII-FL had no effect on malignant cell proliferation in monoculture (n = 3 wells per group). One-way ANOVA. P = 0.42 and P = 0.62, respectively. **b**. In vivo bioluminescent imaging (IVIS) quantification of tumor burden in mice bearing luciferase-expressing SU-DIPG-VI xenografts in the pons, treated with vehicle control or LZP (8 mg kg^−1^) at 14 days following the start of treatment (vehicle, n = 6 mice; LZP, n = 5 mice p = 0.0006). Data are represented as change from baseline luminescence (photon flux). Right, representative IVIS images of xenografted mice treated with LZP or vehicle control. Increased H3K27M^+^ DMG tumor burden is evident in the LZP-treated mice. Two-tailed Student’s t-test. All data are mean ± s.e.m. ***P < 0.001. Source Data

Pharmacological targeting of GABAergic neuron-to-glioma synaptic input using the benzodiazepine lorazepam, a positive allosteric modulator that increases conductance of GABA_A_Rs in the presence of GABA, increased the amplitude of GABAergic postsynaptic currents in DMG cells (Fig. 4d). Treatment of xenografted mice with lorazepam exerted a dose-dependent proliferative effect on H3K27M^+^ DMG in each of three independent patient-derived orthotopic xenograft models (Fig. 4e–g). Although the effect of lorazepam was most robust at high doses (8 mg kg^−1^), in each xenograft model, a significant dose dependency was evident with an analysis of variance (ANOVA) post-test for linear contrast. Benzodiazepines are positive allosteric modulators of GABA_A_Rs that augment GABA signalling, but do not act as direct agonists. Concordantly, the microenvironment of the brain, such as the presence of GABAergic neurons, is required for the proliferative effect of lorazepam: no effect of lorazepam was observed in H3K27M^+^ DMG monocultures, as expected given the lack of GABA-producing cells in the monoculture environment (Extended Data Fig. 7a).

To assess whether this lorazepam-induced increase in malignant cell proliferation was accompanied by an increase in tumour growth, we engineered patient-derived DMG cells to express firefly luciferase, xenografted to the mouse brainstem, and measured tumour burden over time using in vivo bioluminescent imaging. Following a period of engraftment, mice were imaged before initiation of treatment to establish a baseline tumour burden and ensure that mice with equivalent baseline tumour burdens were distributed to the treatment and control groups (baseline mean luminescence was not different between groups, *P* = 0.54 by Student’s *t*-test). Two weeks after beginning administration of vehicle control or high-dose lorazepam, lorazepam-treated mice exhibited a twofold increase in overall tumour burden compared with vehicle-control-treated mice (Extended Data Fig. 7b).

To evaluate the effects of lorazepam on the survival of mice bearing patient-derived DMG xenografted to the brainstem, we administered high-dose lorazepam, low-dose lorazepam or vehicle control over a 4-week period. Mice tolerated both lorazepam dose levels well and began to exhibit morbidity and mortality well after the end of the lorazepam administration period. We found a reduction in survival with high-dose lorazepam administration (Fig. 4h). As with proliferation, a dose-dependent effect on survival was evident with the log-rank test for trend (Fig. 4h). By contrast, and consistent with the negligible GABA-induced currents in H3/IDH WT gliomas, lorazepam did not change glioma proliferation rates in mice bearing patient-derived xenografts of H3/IDH WT hemispheric high-grade glioma (Fig. 4i). Together, these data highlight the glioma-subtype specificity of GABAergic signalling in glioma.

## Discussion

Glutamatergic neuronal activity has emerged as a powerful regulator of glioma progression^2–4,6,30,31^. Across multiple clinically and molecularly distinct forms of paediatric and adult gliomas, activity-regulated paracrine factors such as BDNF and shed neuroligin 3 promote glioma growth^1,2,6,30^. Similarly, AMPAR-mediated glutamatergic synapses drive progression in both H3K27M-altered DMG and hemispheric (H3/IDH WT) GBMs^3,4^. Here we have demonstrated that, akin to its trophic actions on developing neurons^45^, GABAergic signalling also promotes glioma progression through GABAergic synapses that are depolarizing and growth-promoting in the specific disease context of DMGs. Although the structural and electrophysiological evidence here supports the concept of GABAergic neuron-to-glioma synapses, it is important to note that GABA may also induce currents extrasynaptically and through tonic GABA signalling. In contrast to DMG, only minimal currents were found in the hemispheric (IDH/H3 WT) high-grade glioma models used here; as IDH WT HGG is a heterogeneous disease, it is possible that some IDH WT hemispheric gliomas may respond to GABA heterogenously^7^.

The benzodiazepine lorazepam, which potentiates signalling through GABA_A_Rs, increases glioma GABAergic currents, promotes DMG proliferation and growth, and shortens survival in H3K27M-altered DMG models. Lorazepam is commonly used in children with DMG for nausea, anxiety, claustrophobia during MRI scans and other medical procedures, and for other reasons. Although lorazepam is an important medication for palliative care, especially in low doses at end of life, possible risks of high-dose lorazepam in this patient population should be further evaluated in clinical studies. Conversely, and further underscoring differences between DMG and IDH WT hemispheric high-grade gliomas, preclinical studies have indicated that GABA and GABAergic interneurons may instead be growth inhibitory in hemispheric (H3/IDH WT) adult GBM models^44,46^. These findings underscore the therapeutic importance of elucidating the neurophysiology of defined subtypes of brain cancers to identify the patient populations for which a particular neurophysiological drug may be beneficial or detrimental. Understanding the neuroscience of brain tumours will enable the development of effective and safe therapeutic approaches, incorporating neuroscience-informed therapies into combinatorial strategies targeting both cell-intrinsic and microenvironmental mechanisms that drive progression of these devastating cancers.

## Methods

### Human samples and data

For all human tissue and cell studies, informed consent was obtained, and tissue was used in accordance with protocols approved by the Stanford University Institutional Review Board.

### Mice and housing conditions

All in vivo experiments were conducted in accordance with protocols approved by the Stanford University Institutional Animal Care and Use Committee (IACUC) and performed in accordance with institutional guidelines. The IACUC implements regulations from the US Department of Agriculture (USDA), the Public Health Service (PHS) policy, California State Regulations and Stanford University Polices and Guidelines to ensure effective and ethical animal research programmes. Animals were housed according to standard guidelines with free access to food and water in a 12-h light–12-h dark cycle, a temperature of 21 °C and 60% humidity. For brain tumour xenograft experiments, the IACUC does not set a limit on maximal tumour volume but rather on indications of morbidity. In no experiments were these limits exceeded as mice were euthanized if they exhibited signs of neurological morbidity or if they lost 15% or more of their body weight.

### Orthotopic xenografting and allografting

For all xenograft studies, NSG mice (NOD-SCID-IL2Rγ chain-deficient; The Jackson Laboratory) were used. Male and female mice were used equally. A single-cell suspension from cultured SU-DIPG-VI–GFP, SU-DIPG-XIII-FL–GFP, SU-DIPG-50–GFP, SU-pcGBM2–GFP or SU-DIPG-XIII-pons patient-derived glioma cells^3,47^ were prepared in sterile PBS immediately before the xenograft procedure. Animals at postnatal day 28–30 (P28–30) were anaesthetized with 1–4% isoflurane and placed in a stereotactic apparatus. The cranium was exposed via midline incision under aseptic conditions. Approximately 300,000 cells in 3 µl sterile PBS were stereotactically implanted through a 26-gauge burr hole, using a digital pump at an infusion rate of 0.4 µl min^−1^ and 26-gauge Hamilton syringe. For all electrophysiology and optogenetics experiments, cells were implanted into the CA1 region of the hippocampus (1.5 mm lateral to midline, −1.8 mm posterior to bregma and −1.4 mm deep to the cranial surface). SU-DIPG-XIII-FL–GFP and SU-pcGBM2–GFP for lorazepam treatments were xenografted into the premotor cortex (0.5 mm lateral to midline, 1.0 mm anterior to bregma and −1.75 mm deep to the cranial surface). For pontine xenografting, coordinates were 1.0 mm lateral to midline, −0.8 mm posterior to lambda and −5.0 mm deep to the cranial surface. At the completion of infusion, the syringe needle was allowed to remain in place for a minimum of 2 min, then manually withdrawn at a rate of 0.875 mm min^−1^ to minimize backflow of the injected cell suspension. The patient-derived glioma cells used were routinely authenticated using short tandem repeat (STR) fingerprinting to verify identity and lack of contamination.

### In vivo bioluminescence imaging

For in vivo monitoring of tumour growth, bioluminescence imaging was performed using an in vivo bioluminescent imaging system (Xenogen). Mice were orthotopically xenografted with firefly luciferase-expressing H3K27M-mutant DMG cells (SU-DIPG-VI cell xenografted to the pons)^30^. For imaging of tumour burden, mice were placed under isofluorane anaesthesia, injected with luciferin substrate and imaged. Animals were imaged at baseline and randomized based on tumour size by an investigator blinded to experimental conditions, so that experimental groups contained an equivalent range of tumour sizes. All total flux values were then normalized to baseline values to determine the fold change of tumour growth.

### Mouse lorazepam treatment studies

For histological analyses of tumour proliferation, NSG mice were xenografted as above with SU-DIPG-VI–GFP, SU-DIPG-XIII-FL–GFP, SU-DIPG-50–GFP or SU-pcGBM2–GFP cells and randomized to treatment group by an investigator blinded to experimental conditions. Four-to-six weeks post-xenograft, mice were treated with systemic administration of lorazepam (8 mg kg^−1^ or 2 mg kg^−1^ daily; Hospira) via intraperitoneal injection for 4 weeks (5 days per week). Controls were treated with an identical volume of the relevant vehicle.

For analysis of tumour growth, bioluminescence imaging was performed as described above for mice bearing SU-DIPG-VI–GFP-luciferase cells xenografted into the pons before treatment and at 14 days after starting treatment with lorazepam. Tumour burden was assessed as the fold change in change in luminescence from baseline.

For the lorazepam treatment survival study, SU-DIPG-XIII-pons cells were xenografted to the pons as above, and lorazepam was administered (low dose of 2 mg kg^−1^ intraperitoneal, high dose of 8 mg kg^−1^ intraperitoneal, or vehicle control) beginning 3 days after xenografting and administered 5 days a week for 28 days. Morbidity criteria used were either reduction of weight by 15% initial weight or severe neurological motor deficits consistent with brainstem dysfunction (that is, hemiplegia or an incessant stereotyped circling behaviour seen with ventral midbrain dysfunction). Kaplan–Meier survival analysis using log-rank testing was performed to determine statistical significance. Of note, mice tolerated both high-dose and low-dose lorazepam regimens, without becoming over-sedated. With the higher dose (8 mg kg^−1^), mice initially exhibited decreased activity, but appeared to become acclimated to the dose within a couple of days and then behaved normally in their cages. When mice died, which occurred after the 28-day period of lorazepam administration, they exhibited symptoms of increased tumour burden (motor and balance symptoms as typically seen with brainstem tumour disease progression).

### Patient-derived and mouse model-derived cell culture

High-grade glioma cultures SU-DIPG-VI, SU-DIPG-XIII-FL, SU-DIPG-50, SU-pcGBM2 and SU-DIPG-XIII-pons were generated as previously described^13^. In brief, tissue was obtained from high-grade glioma (WHO grade III or IV) tumours at the time of biopsy or from early post-mortem donations in accordance with Institutional Review Board-approved protocols. Tissue was dissociated both mechanically and enzymatically and grown in a defined, serum-free medium designated ‘tumour stem media’, consisting of neurobasal(-A) (Invitrogen), B27(-A) (Invitrogen), human bFGF (20 ng ml^−1^; Shenandoah), human EGF (20 ng ml^−1^; Shenandoah), human PDGF-AA (10 ng ml^−1^) and PDGF-BB (10 ng ml^−1^; Shenandoah) and heparin (2 μg ml^−1^; Stem Cell Technologies). For all patient-derived cultures, mycoplasma testing was routinely performed, and STR DNA fingerprinting was performed every 3 months to verify authenticity. The STR fingerprints and clinical characteristics for the patient-derived cultures and xenograft models used have been previously reported^48^.

### Single-cell sequencing analysis

#### Data curation and preprocessing

We combined publicly available single-cell datasets from ten adult IDH-mutant glioma samples, three adult WT IDH glioma samples, six paediatric H3K27M-mutant DMG samples as well as the single-cell transcriptome of patient-derived SU-DIPG-VI and SU-DIPG-XIII-FL cells^15,32^. For all analyses of these datasets, R studio 1.4.1106-5 was used. Cell filtering was conducted similarly to the original studies, resulting in the retention of 5,096 IDH-mutant glioma malignant cells, 599 adult WT IDH glioma malignant cells and 2,259 paediatric H3K27M^+^ DMG malignant cells.

For most analyses, transcripts per million (TPM) entries in the matrix were normalized using the formula $E={\log }_{2}\left(\frac{{\rm{TPM}}}{10}+1\right)$. The values were divided by 10 because the actual complexity is assumed to be around 100,000 rather than 1 million, as implied by the TPM measure. After normalization, the data were centred by subtracting the mean expression of each gene across all cells.

Before dimensionality reduction, we retained the 7,000 genes with the highest mean expression across all cells in the study. We then computed the UMAP *x* and *y* values using the top 100 principal components (Extended Data Fig. 3).

#### GABAergic synapse-related genes

GABA_A_Rα genes included: *GABRA1*, *GABRA2*, *GABRA3*, *GABRA4*, *GABRA5* and *GABRA6*. GABA_A_Rβ genes included: *GABRB1*, *GABRB2* and *GABRB3*. GABA_A_Rγ genes included: *GABRG1*, *GABRG2* and *GABRG3*.

Total GABA_A_R genes included: *GABRA1*, *GABRA2*, *GABRA3*, *GABRA4*, *GABRA5*, *GABRA6*, *GABRB1*, *GABRB2*, *GABRB3*, *GABRG1*, *GABRG2*, *GABRG3*, *GABRD*, *GABRE*, *GABRP*, *GABRQ*, *GABRR1*, *GABRR2* and *GABRR3*.

Total GABAergic synapse-related genes included: *GABRA1*, *GABRA2*, *GABRA3*, *GABRA4*, *GABRA5*, *GABRA6*, *GABRB1*, *GABRB2*, *GABRB3*, *GABRG1*, *GABRG2*, *GABRG3*, *GABRD*, *GABRE*, *GABRP*, *GABRQ*, *GABRR1*, *GABRR2*, *GABRR3*, *GPHN*, *ARHGEF9*, *NLGN2*, *SLC12A2* and *SLC12A5*.

#### Violin plots

For each sample, we performed first cell-level normalization, and then centred the gene expression around 0 to allow principal component analysis (PCA) computation. Following the PCA reduction, we clustered the cells using shared nearest neighbour clustering. To examine the various GABA_A_R signatures of each of the cells in each cluster, we used the function AddModuleScore by the Seurat package, which calculates the average expression levels of the gene set subtracted by the aggregated expression of 100 randomly chosen control gene sets, where the control gene sets are chosen from matching 25 expression bins corresponding to the tested gene set expression. To calculate the *P* values, we pseudobulked the cells from each sample to generate average gene expression levels per sample using AggregateExpression by the Seurat package. The *P* values were calculated using Wilcoxon rank-sum test by stat_compare_means by the ggpubr package.

#### Differentiation versus stemness analysis

In the H3K27M^+^ DMG dataset^15^, we computed a ‘stemness score’ for each cell. This score was determined by subtracting the higher of either the OC (oligodendrocyte-like) score or the AC (astrocyte-like) score of a cell from its OPC-like score. We also calculated a ‘differentiation score’ for each cell, defined as the maximum of the OC and AC scores. If the AC score was higher, it was multiplied by −1. In cases in which both AC and OC scores were negative, a value of 0 was assigned, with some added jitter. Centring was performed across all samples, and cells were scored based on the genes highlighted in Fig. 1c.

### TagGFP2–GABRG2 cloning and generation of reporter glioma cells

The codon-optimized GABRG2 sequence, with TagGFP2 inserted at the N terminus, was custom synthesized as a gblock (IDT DNA). Subsequently, it was cloned into a lentiviral vector under the *Ef1a* promoter. The resulting plasmid was transformed into Top10 competent cells (Thermo Fisher) and subjected to sequence verification. Following confirmation of the correct sequence, the plasmid was maxiprepped (Qiagen), yielding a concentration ranging from 1 to 2 μg μl^−1^. Once cloned, the plasmid was packaged together with helper plasmids (pΔ8,9 and VSV-g) to generate replication-deficient lentivirus from adherent HEK293T cells (Thermo Fisher). One million target SU-DIPG-VI and SU-DIPG-XIII-FL cells were infected with Lenti-X (Takara) concentrated viral supernatant and allowed to recover for at least 2 weeks.

### Immuno-electron microscopy

Eight weeks after xenografting, mice were euthanized by transcardial perfusion with Karnovsky’s fixative: 2% glutaraldehyde (EMS 16000) and 4% paraformaldehyde (EMS 15700) in 0.1 M sodium cacodylate (EMS 12300), pH 7.4. Transmission electron microscopy was performed in the xenograft tumour mass within the CA1 region of the hippocampus. The samples were post-fixed in 1% osmium tetroxide (EMS 19100) at room temperature for 1 h, washed three times with ultrafiltered water, and then en bloc stained at room temperature for 2 h. The samples were then dehydrated in graded ethanol (50%, 75% and 95%) for 15 min each at 4 °C. The samples were then allowed to equilibrate to room temperature and rinsed in 100% ethanol twice, followed by acetonitrile for 15 min. The samples were infiltrated with EMbed-812 resin (EMS 14120) mixed 1:1 with acetonitrile for 2 h, followed by EMbed-812 mixed 2:1 with acetonitrile for 2 h, and then in Embed-812 for 2 h. The samples were then placed into TAAB capsules with fresh resin and kept overnight at 65 °C. Sections between 40 nm and 60 nm were taken on an Ultracut S (Leica) and mounted on 100-mesh Ni grids (EMS FCF100-Ni). For immunohistochemistry, microetching was done with 10% periodic acid and eluting of osmium with 10% sodium metaperiodate for 15 min at room temperature on parafilm. Grids were rinsed with water three times, followed by 0.5 M glycine quench, and then incubated in blocking solution (0.5% BSA and 0.5% ovalbumin in PBST) at room temperature for 20 min. Primary rabbit anti-GFP (1:300; MBL International) was diluted in the same blocking solution and incubated overnight at 4 °C. The following day, grids were rinsed in PBS three times, and incubated in secondary antibody (1:10 10-nm gold-conjugated IgG TED Pella15732) for 1 h at room temperature and rinsed with PBST followed by water. For each staining set, secondary-only staining was simultaneously performed to control for any nonspecific binding. Grids were contrast stained for 30 s in 3.5% uranyl acetate in 50% acetone followed by staining in 0.2% lead citrate for 90 s. Samples were imaged using a JEOL JEM-1400 transmission electron microscopy (TEM) at 120 kV and images were collected using a Gatan Orius digital camera.

Sections of hippocampal xenografts of SU-DIPG-VI and SU-DIPG-XIII-FL with GFP-tagged GABRG2 were imaged using TEM imaging. In total, 78 images of SU-DIPG-VI xenografts from 4 mice and 137 images of SU-DIPG-XIII-FL from 3 mice were assessed. Electron microscopy images were taken at ×60,000, ×80,000 or ×12,000. GABAergic neuron-to-glioma synapses were defined as those with unequivocal identification of immunogold particle labelling with three or more particles adjacent to the postsynaptic membrane, in addition to the presence of synaptic vesicle clusters and a visually apparent synaptic cleft.

### Neuron-glioma co-culture experiments

Neurons were isolated from the cortices of P0 Sprague-Dawley rat pups of either sex using the Neural Tissue Dissociation Kit–Postnatal Neurons (Miltenyi) and ACK Lysing Buffer (Gibco), followed by the Neuron Isolation Kit, Mouse (Miltenyi) per the manufacturers’ instructions. After isolation, 80,000 neurons were plated onto circular glass coverslips (Electron Microscopy Services) pre-treated for 1 h at 37 °C with poly-l-lysine (Sigma) and then 2 h at 37 °C with 5 µg ml^−1^ mouse laminin (Thermo Fisher). Neurons were cultured in BrainPhys Neuronal Medium (StemCell Technologies) supplemented with B-27 (Invitrogen), 1× GlutaMAX (Invitrogen), penicillin–streptomycin (Invitrogen), 55 µM 2-mercaptoethanol (Gibco), GDNF (5 ng ml^−1^; Shenandoah), BDNF (10 ng ml^−1^; Shenandoah) and TRO19622 (5 µM; Tocris). Half of the medium was replenished on 1 and 3 days in vitro. On 5 days in vitro, half of the medium was replaced in the morning. In the afternoon, 60,000 glioma cells expressing TagGFP2–GABRG2 were plated onto the neurons in half of the conditioned medium. Glioma cells were cultured with neurons for 3 days, and then fixed with 4% paraformaldehyde for 15 min at room temperature and stained for immunofluorescence analysis as described below. The patient-derived glioma cells used were routinely authenticated using STR fingerprinting to verify identity and lack of contamination.

### Slice preparation for electrophysiology

Coronal slices (300 µm thick) containing the hippocampal region were prepared from mice (at least 8 weeks after xenografting) in accordance with a protocol approved by Stanford University IACUC. After rapid decapitation, the brain was removed from the skull and immersed in ice‐cold slicing artificial cerebrospinal fluid (ACSF) containing: 125 mM NaCl, 2.5 mM KCl, 25 mM glucose, 25 mM NaHCO_3_, 1.25 mM NaH_2_PO_4_, 3 mM MgCl_2_ and 0.1 mM CaCl_2_. After cutting, slices were incubated for 30 min in warm (30 °C) oxygenated (95% O_2_ and 5% CO_2_) recovery ACSF containing: 100 mM NaCl, 2.5 mM KCl, 25 mM glucose, 25 mM NaHCO_3_, 1.25 mM NaH_2_PO_4_, 30 mM sucrose, 2 mM MgCl_2_ and 1 mM CaCl_2_ before being allowed to equilibrate at room temperature for an additional 30 min.

### Electrophysiology

Slices were transferred to a recording chamber and perfused with oxygenated, warmed (28–30 °C) recording ACSF containing: 125 mM NaCl, 2.5 mM KCl, 25 mM glucose, 25 mM NaHCO_3_, 1.25 mM NaH_2_PO_4_, 1 mM MgCl_2_ and 2 mM CaCl_2_. NBQX (10 µM) was perfused with the recording ACSF to prevent AMPAR-mediated currents in synaptic response experiments. Tetrodotoxin (0.5 µM) was perfused with the recording ACSF during GABA puff experiments to prevent neuronal action potential firing. Cells were visualized using a microscope equipped with DIC optics (Olympus BX51WI). Recording patch pipettes were filled with CsCl-based pipette solution containing: 150 mM CsCl, 5 mM EGTA, 1 mM MgCl_2_, 10 mM HEPES, 2 mM ATP and 0.3 mM GTP, pH 7.3. Patch electrodes had resistances of 4–5 MΩ. Pipette solution additionally contained Alexa 568 (50 μM) to visualize the cell through dye filling during whole-cell recordings, and recorded slices were post-fixed and stained to confirm cell type. Gramicidin A (60 μg ml^−1^) was added to the pipette solution for perforated patch recordings. A perforated patch was considered satisfactory when an access resistance of approximately 30 MΩ was obtained about 30 min after attaining gigaseal resistance. A leak of Alexa 568 dye from the pipette into the cell during perforated patch recordings indicated a damaged membrane, and the data from such recordings were discarded. *E*_GABA_ was calculated for each individual cell and averaged. In the negative chloride load experiments and in neuronal recordings, recording patch pipettes were filled with CsMe-based pipette solution containing: 135 mM CsMeSO_4_, 12 mM HEPES, 8 mM NaCl, 0.25 mM EGTA, 2 mM MgCl_2_, 2 mM ATP, 0.3 mM GTP and 5 mM phosphocreatine, pH 7.3. Glioma cells were voltage clamped at a holding potential of −70 mV. For all whole-cell patch clamp experiments, series resistance was less than 30 MΩ. Synaptic responses were evoked with a bipolar electrode connected to an Iso-flex stimulus isolator (A.M.P.I.) placed 100–200 μm from the patched xenografted cells. A low-intensity stimulation, sufficient to evoke consistent responses but not higher, was used at a frequency of 0.1 Hz. GABA (1 mM) in recording ACSF was applied via a puff pipette, which was placed approximately 100 μm away from the patched cell and controlled by a Picospritzer II (Parker Hannifin Corp.). Optogenetic stimulation of interneurons was achieved with a 598-nm LED using a pE-4000 illumination system (CoolLED). Optogenetic stimulation of interneurons in acute hippocampus slices was performed with 5-ms pulses of blue light, in the absence of inhibitors such as NBQX. Signals were acquired with a MultiClamp 700B amplifier (Molecular Devices) and digitized at 10 kHz with an Axon Digidata 1550B (Molecular Devices) or an InstruTECH LIH 8 + 8 data acquisition device (HEKA). Data were recorded and analysed using pClamp 11 software suite (Molecular Devices), AxoGraph X (AxoGraph Scientific) and/or IGOR Pro 8 (Wavemetrics). For representative traces, stimulus artefacts preceding the synaptic responses have been removed for clarity. Intracellular chloride concentration was calculated using the Nernst equation. Rise time, decay time and synaptic delay were measured from responses to stimulation in SU-DIPG-VI cells in the presence of NBQX. Rise time was calculated as the time between 10% and 90% of the peak response, as measured from the discernible onset of the averaged response for each cell. Decay time was defined as the monoexponential decay time constant for the averaged response for each cell. Synaptic delay was measured by the time from the end of the 0.5-ms stimulus to the visually discernible beginning of the response.

### Pharmacological agents

Drugs and toxins used for electrophysiology were picrotoxin (50 µM; Tocris), tetrodotoxin (0.5 µM; Tocris), NBQX (10 µM; Tocris), D-AP5 (100 µM; Tocris), bicuculline (10 μM, Tocris), bumetanide (10 µM or 100 μM; Tocris) and lorazepam (10 μM; Hospira). When used for in vitro slice application, drugs were made up as a stock in distilled water or dimethylsulfoxide (DMSO) and dissolved to their final concentrations in ACSF before exposure to slices. The final concentration of DMSO was less than 1%.

### Viral injection and fibre optic placement

Animals were anaesthetized with 1–4% isoflurane and placed in a stereotaxic apparatus. For optogenetic stimulation experiments, 1 µl of AAV8-DLX5/6–ChRmine::mCherry^49^ (virus titre of 1.19 × 10^12^; a gift from K. Deisseroth at Stanford University) was unilaterally injected using Hamilton Neurosyringe and Stoelting stereotaxic injector over 5 min. The viral vector was injected into the hippocampal CA1 region in the right hemisphere at coordinates: 1.5 mm lateral to midline, −1.8 mm posterior to bregma and −1.3 mm deep to the cranial surface. Two weeks following the viral injection, SU-DIPG-XIII-FL cells were xenografted as described above. After 7 weeks of tumour engraftment, an optic ferrule was placed above the CA1 of the hippocampus of the right hemisphere at: 1.5 mm lateral to midline, −1.8 mm posterior to bregma and −1.25 mm deep to the cranial surface.

### Optogenetic stimulation

Optogenetic stimulations were performed at least 10 weeks after the viral vector delivery, 8 weeks after xenografts and 1 week after optic ferrule implantation. Freely moving animals were connected to a 595-nm fibre-coupled LED laser system with a monofibre patch cord. Optogenetic stimulation was performed with cycles of 595-nm light pulses at 40 Hz frequency, 10-ms light pulse width and a light power output of 10–15 mW from the tip of the optic fibre, which lasted for 30 s, followed by 90 s recovery over a 30-min period. Animals were injected intraperitoneally with 40 mg kg^−1^ EdU (5-ethynyl-2′-deoxyuridine; E10187, Invitrogen) before the session, and were perfused 24 h after optogenetic stimulation for proliferation assays and 90 min after optogenetic stimulation for Fos expression in interneurons.

### Perfusion and immunohistochemistry

Animals were anaesthetized with intraperitoneal avertin (tribromoethanol), then transcardially perfused with 20 ml of PBS. Brains were fixed in 4% paraformaldehyde overnight at 4 °C and then transferred to 30% sucrose for cryoprotection. Brains were then embedded in Tissue-Tek O.C.T. (Sakura) and sectioned in the coronal plane at 40 µm using a sliding microtome (Microm HM450; Thermo Scientific).

For immunohistochemistry, coronal sections were incubated in blocking solution (3% normal donkey serum, 0.3% Triton X-100 in TBS) at room temperature for 1–2 h. Chicken anti-GFP (1:500; Abcam), mouse anti-human nuclei clone 235-1 (1:100; Millipore), rabbit anti-Ki67 (1:500; Abcam), guinea pig anti-VGAT (1:500; Synaptic Systems), mouse anti-gephyrin (1:500; Synaptic Systems), rabbit anti-GAD65 (1:500; Abcam), mouse anti-GAD67 (1:500; Millipore Sigma), rabbit anti-H3K27M (1:500; Millipore) or mouse anti-Fos (1:500; Santa Cruz Biotechnology) were diluted in antibody diluent solution (1% normal donkey serum in 0.3% Triton X-100 in TBS) and incubated 24–36 h at 4 °C. Sections were then rinsed three times in TBS and incubated in secondary antibody solution containing Alexa 488 donkey anti-chicken IgG, Alexa 488 donkey anti-rabbit IgG, Alexa 594 donkey anti-mouse IgG, Alexa 594 donkey anti-rabbit IgG, Alexa 647 donkey anti-mouse IgG, Alexa 647 donkey anti-rabbit IgG and DyLight 405 AffiniPure donkey anti-guinea pig IgG used at 1:500 (Jackson Immuno Research) in antibody diluent at 4 °C for 2 h. Sections were rinsed three times in TBS and mounted with ProLong Gold Mounting medium (Life Technologies).

Human autopsy sample immunohistochemistry for H3K27M antigen was performed on 5-µm sections of FFPE samples in the Stanford University clinical pathology laboratory according to CLIA-certified protocols and counterstained with haemotoxylin.

### Immunocytochemistry

For immunocytochemistry, fixed coverslips were incubated in blocking solution (3% normal donkey serum and 0.3% Triton X-100 in TBS) at room temperature for 30 min. Primary antibodies chicken anti-neurofilament (H+M; 1:200; Aves Labs), guinea pig anti-synapsin1/2 (1:500; Synaptic Systems), mouse anti-nestin (1:500; Abcam) and rabbit anti-GFP (1:500; Novus Biologicals) were diluted in the antibody diluent solution (1% normal donkey serum in TBS) and incubated overnight at 4 °C. Coverslips were washed three times with TBS and incubated with secondary antibodies Alexa 594 donkey anti-chicken IgY (1:500; Jackson ImmunoResearch), DyLight 405 donkey anti-guinea pig IgG (1:500; Jackson ImmunoResearch), Alexa 647 donkey anti-mouse IgG (1:1,000; Jackson ImmunoResearch) and Alexa 488 donkey anti-rabbit IgG (1:1,000; Jackson ImmunoResearch) in the antibody diluent solution for 45 min at room temperature. Coverslips were washed three times with TBS and mounted with ProLong Gold Antifade Mountant (Invitrogen).

### Confocal imaging

Images were acquired using a ×63 oil immersion objective (synaptic puncta imaging), ×40 oil immersion objective or ×20 air objective of a Zeiss LSM700, Zeiss LSM800 or Zeiss LSM980 scanning confocal microscope and Zen imaging software (v8.1; Carl Zeiss). 3D image reconstructions were processed using IMARIS (v10.0.0) software. Cell quantification within xenografts was performed by an investigator blinded to experimental conditions.

### Quantification of cell proliferation

Confocal images were analysed and quantified using ImageJ (v2.1.0/1.53c). For Ki67 analysis, 3 fields for quantification were selected from each of 3 consecutive sections in a 1-in-6 series of 40-μm coronal sections with respect to overall tumour burden. Within each field, all human nuclear antigen (HNA)-positive and GFP-positive tumour cells were quantified to determine tumour burden within the areas quantified. HNA-positive cells were then assessed for co-labelling with Ki67. To calculate the proliferation index (the percentage of proliferating tumour cells for each mouse), the total number of HNA-positive cells co-labelled with Ki67 across all areas quantified was divided by the total number of cells counted across all areas quantified (Ki67^+^/HNA^+^).

### Unbiased stereology

GABAergic interneurons immunopositive for GAD67 were visualized with a MBF Zeiss Axiocam light microscope. Only those cells for which GAD67 unequivocally marked the soma were counted. HNA was used to mark xenografted patient-derived DMG cells. Cell numbers were determined through unbiased stereology using Stereo Investigator software (MBF Bioscience, version 2023). Frontal cortex regions of interest were defined in sections with a visible corpus callosum by drawing a vertical line from the tip of the cingulum to the brain surface, and a horizontal line from this point towards the midline. The end point of these lines was then connected along the cortical edge to delineate the frontal cortex, including the medial prefrontal cortex and the motor cortex. This method was consistently applied across all sections. Sections were traced at ×2.5 magnification and images were acquired at ×40 from 1 in 6 serial sections throughout the region of interest (3–4 sections per animal). Stereological parameters were determined ensuring that at least 100–300 cells would be counted per animal and the Gunderson *m* = 1 coefficient of error was less than 0.1. The sampling grid size was set to 250 × 250 μm; the counting frame was set to 100 × 100 μm. Exposure time was kept uniform across all samples imaged within each experiment.

### EdU incorporation assay

DMG tumour neurosphere cultures SU-DIPG-VI, SU-DIPG-XIII-FL and SU-DIPG-50 were generated as previously described^3,7^ from early post-mortem tissue donations and grown as tumour neurospheres in defined, serum-free tumour stem media, consisting of 1:1 mixture of neurobasal(-A) (Invitrogen) and D-MEM/F-12 (Invitrogen), HEPES buffer (Invitrogen), MEM sodium pyruvate (Invitrogen), MEM non-essential amino acids (Invitrogen), GlutaMAX-1 supplement (Invitrogen), B27(-A) (Invitrogen), human bFGF (20 ng ml^−1^; Shenandoah), human EGF (20 ng ml^−1^; Shenandoah), human PDGF-AA (10 ng ml^−1^; Shenandoah), PDGF-BB (10 ng ml^−1^; Shenandoah) and heparin (2 ng ml^−1^; Stem Cell Technologies).

One hundred thousand glioma cells were plated onto circular glass coverslips (Electron Microscopy Services) pre-treated for 1 h at 37 °C with poly-l-lysine (Sigma) and then 1 h at 37 °C with 10 µg ml^−1^ natural mouse laminin (Thermo Fisher). Dimethyl sulfoxide (Sigma-Aldrich) or drugs at the concentrations indicated (dissolved in dimethyl sulfoxide) were added to the coverslips. EdU (10 μM) was added to each coverslip. Cells were fixed after 24 h using 4% paraformaldehyde in PBS and stained using the Click-iT EdU kit and protocol (Invitrogen). The proliferation index was then determined by quantifying the fraction of EdU-labelled cells to DAPI-labelled cells using confocal microscopy.

### Statistical analyses

Statistical tests were conducted using Prism (v9.1.0; GraphPad) software. Gaussian distribution was confirmed by the Shapiro–Wilk normality test. For parametric data, unpaired two-tailed Student’s *t*-tests or one-way ANOVA with Dunnett’s post-hoc test to examine pairwise differences and/or test for linear contrast were used as indicated in figure legends. Paired two-tailed Student’s *t*-tests or repeated measures one-way ANOVA with Dunnett’s post-hoc analysis were used in electrophysiological experiments within the same cell. Simple linear regression analysis was used to determine the *x* intercept in the current–voltage relationship experiments. Two-tailed log-rank analyses were used to analyse statistical significance of Kaplan–Meier survival curves. Statistical test results are reported in the figure legends and in Supplementary Table 1. At least three mice for in vivo experiments and at least three independent coverslips for in vitro experiments were used per test group to attain 80% power to detect an effect size of 20% at a significance level of 0.05. Statistical analyses of retrospective patient data are described above.

### Reporting summary

Further information on research design is available in the Nature Portfolio Reporting Summary linked to this article.

## Online content

Any methods, additional references, Nature Portfolio reporting summaries, source data, extended data, supplementary information, acknowledgements, peer review information; details of author contributions and competing interests; and statements of data and code availability are available at 10.1038/s41586-024-08579-3.

## Supplementary information

Supplementary Table 1

Reporting Summary

Supplementary Video 1 GABAergic synapse in neuron-glioma co-culture Three-dimensional reconstruction of confocal micrograph illustrating GABAergic neuron-to-glioma synaptic puncta co-localization in neuron+glioma co-cultures. Patient-derived DMG cells (SU-DIPGXIII-FL; nestin, blue) expressing GABRG2-GFP (green, postsynaptic) co-localizes with the presynaptic marker synapsin (white) on rat hippocampal neurons (neurofilament, red).

## Source data

Source Data Figs. 3, 4 and Source Data Extended Data Figs. 4–7

## Data Availability

Single-cell RNA-seq data were analysed from publicly available datasets on the Gene Expression Omnibus (GEO): accession numbers GSE102130 and GSE134269. Data for all other experiments in the Article can be found in the Source Data file. Source data are provided with this paper.

## Code availability

Sources for all code used have been provided; no custom code was created for this study.
