# Supplementary material for: GABAergic neuron-to-glioma synapses in diffuse midline gliomas
Source: Nature. 2025 Feb 19;639(8056):1060–8. doi: 10.1038/s41586-024-08579-3 (PMC11946904; doi:10.1038/s41586-024-08579-3)
Supplement: Supplementary file 1 — Supplementary Table 1 [file 41586_2024_8579_MOESM1_ESM.docx]

**GABAergic neuron-to-glioma synapses in diffuse midline gliomas**

Tara Barron^1^, Belgin Yalçın^1^, Minhui Su^1^, Youkyeong Gloria Byun^1,2^, Avishai Gavish^1^, Kiarash Shamardani^1^, Haojun Xu^1^, Lijun Ni^1^, Neeraj Soni^1,2^, Vilina Mehta^1^, Samin Maleki Jahan^1^, Yoon Seok Kim^1^, Kathryn R. Taylor^1^, Michael B. Keough^1^, Michael A. Quezada^1^, Anna C. Geraghty^1,^ Rebecca Mancusi^1^, Linh Thuy Vo^1^, Enrique Herrera Castañeda^1^, Pamelyn J. Woo^1^, Claudia K. Petritsch^3^, Hannes Vogel^4^, Kai Kaila^5,6,^, Michelle Monje^1,2,3,4*^

1 Department of Neurology and Neurological Sciences, Stanford University, Stanford CA 94305 USA

2 Howard Hughes Medical Institute, Stanford University, Stanford CA 94305 USA

3 Department of Neurosurgery, Stanford University, Stanford CA 94305 USA

4 Department of Pathology, Stanford University, Stanford CA 94305 USA

5 Faculty of Bio- and Environmental Sciences (MIBS), University of Helsinki, Finland

6 Neuroscience Center (HiLIFE), University of Helsinki, Finland

*Please send correspondence to: Michelle Monje MD PhD (mmonje@stanford.edu)

**Table of Contents**

Supplementary Table 1: Additional Statistical Information………………………………1

Supplementary Video 1: GABAergic synapse in neuron-glioma co-culture……………...3

**Supplementary Table 1: Additional Statistical Information.**

| **Panel** | **Statistical test** | **Sample size** | **P value** | **Test statistic** | **95% confidence interval** |
| --- | --- | --- | --- | --- | --- |
| Figure 3f | Two-tailed paired Student’s t-test | n = 7 cells from 5 mice | P=0.0341 | t=2.731, df=6 | -155.1 to -8.510 |
| Figure 3g | Repeated measures one-way ANOVA with Dunnett’s post hoc analysis | n = 4 cells from 3 mice | Control vs D-AP5 P=0.1787; Control vs NBQX P=0.1093; Control vs Bic P=0.0287 | F=28.78 | Control vs D-AP5 -12.51 to 47.83; Control vs NBQX -8.518 to 54.64; Control vs Bic 17.88 to 172.7 |
| Figure 4b | Two-tailed paired Student’s t-test | n = 3 cells from 3 mice | P=0.0075 | t=11.46, df=2 | 15.79 to 34.77 |
| Figure 4d | Two-tailed unpaired Student’s t-test | mock stim, n = 6 mice; stim, n = 8 mice | P=0.0386 | t=2.322, df=12 | 0.002243 to 0.07062 |
| Figure 4e | One-way ANOVA with Dunnett's post hoc analysis and post-test for linear contrast | vehicle, n = 7 mice; low dose, n = 8 mice; high dose, n = 7 mice | Dunnett's post hoc: Vehicle vs LZP (low) P=0.4933, Vehicle vs LZP (high) P=0.0228; Linear contrast: P=0.0124 | F=3.926 | Dunnett's post hoc: Vehicle vs LZP (low) -0.07607 to 0.03018, Vehicle vs LZP (high) -0.1184 to -0.008693; Linear contrast: 0.007713 to 0.05585 |
| Figure 4f | One-way ANOVA with Dunnett's post hoc analysis and post-test for linear contrast | vehicle, n = 7 mice; low dose, n = 9 mice; high dose, n = 8 mice | Dunnett's post hoc: Vehicle vs LZP (low) P=0.325, Vehicle vs LZP (high) P=0.033; Linear contrast: P=0.0181 | F=3.287 | Dunnett's post hoc: Vehicle vs LZP (low) -0.07882 to 0.02222, Vehicle vs LZP (high) -0.1081 to -0.004375; Linear contrast: 0.005314 to 0.05094 |
| Figure 4g | One-way ANOVA with Dunnett's post hoc analysis and post-test for linear contrast | vehicle, n = 8 mice; low dose, n = 8 mice; high dose, n = 6 mice | Dunnett's post hoc: Vehicle vs LZP (low) P=0.1017, Vehicle vs LZP (high) P=0.0041; Linear contrast: P=0.0020 | F=6.389 | Dunnett's post hoc: Vehicle vs LZP (low) -0.1135 to 0.009312, Vehicle vs LZP (high) -0.1646 to -0.03198; Linear contrast: 0.02041 to 0.07818 |
| Figure 4i | One-way ANOVA with Dunnett's post hoc analysis | vehicle, n = 4 mice; low dose, n = 3 mice; high dose, n = 4 mice | Dunnett's post hoc: Vehicle vs LZP (low) P=0.8637, Vehicle vs LZP (high) P=0.732 | F=0.2463 | Dunnett's post hoc: Vehicle vs LZP (low) -0.07107 to 0.1005, Vehicle vs LZP (high) -0.05918 to 0.09965 |
| Extended Data Figure 6c | Two-tailed paired Student’s t-test | SU-DIPGVI: n = 4 mice per group; SU-DIPGXIII-FL: n = 3 mice per group | SU-DIPGVI: P=0.1147; SU-DIPGXIII-FL: P=0.829 | SU-DIPGVI:  t=2.204, df=3; SU-DIPGXIII-FL:  t=0.2455, df=2 | SU-DIPGVI:  -336.3 to 1852; SU-DIPGXIII-FL:  -1814 to 1618 |
| Extended Data Figure 6d | Two-tailed paired Student’s t-test | n = 5 mice per group | P=0.0078 | t=4.936, df=4 | 13.90 to 49.65 |
| Extended Data Figure 6e | Two-tailed unpaired Student’s t-test | n = 3 mice per group | P=0.0264 | t=3.435, df=4 | 7.595 to 71.60 |
| Extended Data Figure 7a | One-way ANOVA | n = 3 wells per group | SU-DIPGVI: P=0.4200; SU-DIPGXIII-FL: P=0.6182 | SU-DIPGVI: F=1.055; SU-DIPGXIII-FL: F=0.6259 | N/A |
| Extended Data Figure 7b | Two-tailed unpaired Student’s t-test | vehicle, n = 6 mice; LZP (high dose), n = 5 mice | P=0.0006 | t=5.132, df=9 | 1.532 to 3.948 |

**Supplementary Video 1: GABAergic synapse in neuron-glioma co-culture**

Three-dimensional reconstruction of confocal micrograph illustrating GABAergic neuron-to-glioma synaptic puncta co-localization in neuron+glioma co-cultures. Patient-derived DMG cells (SU-DIPGXIII-FL; nestin, blue) expressing GABRG2-GFP (green, postsynaptic) co-localizes with the presynaptic marker synapsin (white) on rat hippocampal neurons (neurofilament, red).
