# Supplementary material for: GABAergic neuron-to-glioma synapses in diffuse midline gliomas
Source: Nature. 2025 Feb 19;639(8056):1060–8. doi: 10.1038/s41586-024-08579-3 (PMC11946904; doi:10.1038/s41586-024-08579-3)
Supplement: Supplementary file 2 — Reporting Summary [file 41586_2024_8579_MOESM2_ESM.pdf]

nature portfolio

Corresponding author(s): Michelle Monje

Last updated by author(s): 12/2/2024

Reporting Summary

Nature Portfolio wishes to improve the reproducibility of the work that we publish. This form provides structure for consistency and transparency in reporting. For further information on Nature Portfolio policies, see our Editorial Policies and the Editorial Policy Checklist.

Statistics

For all statistical analyses, confirm that the following items are present in the figure legend, table legend, main text, or Methods section.

n/a	Confirmed	
☐	☒	The exact sample size (*n*) for each experimental group/condition, given as a discrete number and unit of measurement
☐	☒	A statement on whether measurements were taken from distinct samples or whether the same sample was measured repeatedly
☐	☒	The statistical test(s) used AND whether they are one- or two-sided<br>*Only common tests should be described solely by name; describe more complex techniques in the Methods section.*
☐	☒	A description of all covariates tested
☐	☒	A description of any assumptions or corrections, such as tests of normality and adjustment for multiple comparisons
☐	☒	A full description of the statistical parameters including central tendency (e.g. means) or other basic estimates (e.g. regression coefficient) AND variation (e.g. standard deviation) or associated estimates of uncertainty (e.g. confidence intervals)
☐	☒	For null hypothesis testing, the test statistic (e.g. *F*, *t*, *r*) with confidence intervals, effect sizes, degrees of freedom and *P* value noted<br>*Give P values as exact values whenever suitable.*
☒	☐	For Bayesian analysis, information on the choice of priors and Markov chain Monte Carlo settings
☒	☐	For hierarchical and complex designs, identification of the appropriate level for tests and full reporting of outcomes
☐	☒	Estimates of effect sizes (e.g. Cohen's *d*, Pearson's *r*), indicating how they were calculated

Our web collection on statistics for biologists contains articles on many of the points above.

Software and code

Policy information about availability of computer code

Data collection	Electrophysiology data were collected with a MultiClamp 700B amplifier (Molecular Devices) and digitized at 10 kHz with an Axon Digidata 1550B (Molecular Devices) or an InstruTECH LIH 8+8 data acquisition device (HEKA). Data were recorded and analyzed using pClamp 11 software suite (Molecular Devices), AxoGraph X (AxoGraph Scientific) and/or IGOR Pro 8 (Wavemetrics). Confocal images were acquired on either a Zeiss LSM700, Zeiss Airyscan1 LSM800, or Zeiss Airyscan2 LSM980 using Zen 2011 v8.1. Bioluminescence imaging was performed using an IVIS imaging system (Xenogen).
Data analysis	Statistical tests were conducted using GraphPad Prism v9.1.0 software. For analysis of previously published scRNAseq data, R studio 1.4.1106-5 was used, and the Seurat package was used for violin plot construction. Confocal images were analyzed and quantified using ImageJ v.2.1.0/1.53c. 3D image reconstructions were processed using IMARIS v10.0.0.

For manuscripts utilizing custom algorithms or software that are central to the research but not yet described in published literature, software must be made available to editors and reviewers. We strongly encourage code deposition in a community repository (e.g. GitHub). See the Nature Portfolio guidelines for submitting code & software for further information.

Data

Policy information about availability of data

All manuscripts must include a data availability statement. This statement should provide the following information, where applicable:

- Accession codes, unique identifiers, or web links for publicly available datasets
- A description of any restrictions on data availability
- For clinical datasets or third party data, please ensure that the statement adheres to our policy

All unique materials such as patient-derived cell cultures are freely available and can be obtained by contacting the corresponding author with a standard MTA with

Stanford University. Single cell RNAseq data were analyzed from publicly available datasets on GEO (GSE102130 and GSE134269). Data for all figures in the manuscript can be found in the Source Data.

Field-specific reporting

Please select the one below that is the best fit for your research. If you are not sure, read the appropriate sections before making your selection.

☒ Life sciences ☐ Behavioural & social sciences ☐ Ecological, evolutionary & environmental sciences

For a reference copy of the document with all sections, see nature.com/documents/nr-reporting-summary-flat.pdf

Life sciences study design

All studies must disclose on these points even when the disclosure is negative.

Sample size	At least three mice for in vivo experiments, and at least three independent coverslips for in vitro experiments, were used per test group to attain 80% power to detect an effect size of 20% at significance level 0.05.
Data exclusions	No data were excluded from analyses.
Replication	The number and type of biological replicates is indicated in the figure legends and was three or greater for all experiments. At least three independent coverslips or at least three mice were used for each experiment.
Randomization	Mice were randomly assigned to treatment or control groups for all lorazepam experiments. Mice were randomly assigned to stimulation or unstimulated/mock stimulation groups for all optogenetics experiments.
Blinding	Imaging and quantification of histological and IVIS experiments were performed by blinded experimenters. Quantification of electrophysiological data was performed within the same cell before and after treatment and therefore not blinded.

Reporting for specific materials, systems and methods

We require information from authors about some types of materials, experimental systems and methods used in many studies. Here, indicate whether each material, system or method listed is relevant to your study. If you are not sure if a list item applies to your research, read the appropriate section before selecting a response.

Materials & experimental systems

n/a	Involved in the study	
☐	☒	Antibodies
☐	☒	Eukaryotic cell lines
☒	☐	Palaeontology and archaeology
☐	☒	Animals and other organisms
☐	☒	Human research participants
☒	☐	Clinical data
☒	☐	Dual use research of concern

Methods

n/a	Involved in the study	
☒	☐	ChIP-seq
☒	☐	Flow cytometry
☒	☐	MRI-based neuroimaging

Antibodies

Antibodies used

Primary antibodies used in immunohistochemistry: chicken anti-GFP (1:500, ab1397, Abcam), mouse anti-human nuclei clone 235-1 (1:100, MAB1281, Millipore), rabbit anti-Ki67 (1:500, ab15580, Abcam), guinea pig anti-VGAT (1:500, 131 004, Synaptic Systems), mouse anti-gephyrin (1:500, 147 001, Synaptic Systems), rabbit anti-GAD65 (1:500, Ab239372, Abcam), mouse anti-GAD67 (1:500, MAB5406, Millipore Sigma), rabbit anti-H3K27M (1:500, ABE419, Millipore), and mouse anti-cFos (1:500, sc-166940, Santa Cruz Biotechnology).

Primary antibodies used in immunocytochemistry: chicken anti-Neurofilament-H (1:200, NFH, Aves Labs), chicken anti-Neurofilament-M (1:200, NFM, Aves Labs), guinea pig anti-synapsin (1:500, 106-004, Synaptic Systems), mouse anti-nestin (1:100, ab10538, Abcam), and rabbit anti-GFP (1:500, NB600-308, Novus Biologicals).

Secondary antibodies used in immunohistochemistry: Alexa 488 donkey anti-chicken IgG (703-545-155), Alexa 488 donkey anti-rabbit IgG (711-545-152), Alexa 594 donkey anti-mouse IgG (715-585-150), Alexa 594 donkey anti-rabbit IgG (711-585-152), Alexa 647 donkey anti-mouse IgG (715-605-151), Alexa 647 donkey anti-rabbit IgG (711-605-152), DyLight™ 405 AffiniPure donkey anti-guinea pig IgG (706-475-148) at 1:500 (Jackson ImmunoResearch).

Secondary antibodies used in immunocytochemistry: Alexa 594 donkey anti-chicken IgY (703-585-155), DyLight 405 donkey anti-guinea pig IgG (706-475-148), Alexa 647 donkey anti-mouse IgG (715-605-151), and Alexa 488 donkey anti-rabbit IgG (711-545-152) at 1:500 (Jackson ImmunoResearch).

Validation	All antibodies have been validated either in the literature and/or by manufacturers for use in mouse immunohistochemistry. To further validate the antibodies in our hands, we confirmed each antibody had staining in the expected cellular patterns and brain-wide distributions.<br>Antibody validation information: chicken anti-GFP (ab13970, Abcam; Reactivity: Species independent. Manufacturer validation: ICC: GFP-transfected NIH/3T3 Mouse embryo fibroblast cell line. Publication Figure: Figures 2d-e and 3c, Extended Data Figures 4b and 6e, IF of patient-derived glioma xenograft), mouse anti-human nuclei clone 235-1 (MAB1281, Millipore; Reactivity: human only, Manufacturer validation: IF neural stem cells transplanted into rat brain. Publication Figure: Figures 3c, 4c, 4g, 4i, IF of patient-derived glioma xenograft), rabbit anti-Ki67 (ab15580, Abcam; Reactivity: mouse, human. Manufacturer validation: IF in Mef1 and HeLa cultures, human skin tissue. Publication Figure: Figures 4g, 4i, IF of patient-derived glioma xenograft), guinea pig anti-VGAT (131 004, Synaptic Systems; Reactivity: rat, mouse, zebrafish, ape. Manufacturer validation: ICC of rat hippocampal neurons. Publication Figure: Figure 2d-e, Extended Data Figure 4b, IF of patient-derived glioma xenograft), mouse anti-gephyrin (147 011, Synaptic Systems; Reactivity: human, rat, mouse, pig, goldfish, zebrafish, chicken. Manufacturer validation: ICC of rat hippocampal neurons and IF in mouse spinal cord. Publication Figure: Figure 2d-e, Extended Data Figure 4b, IF of patient-derived glioma xenograft), rabbit anti-GAD65 (Ab239372, Abcam; Reactivity: mouse, rat. Manufacturer validation: IF of mouse cerebellum. Publication Figure: Extended Data Figure 6a, IF of patient-derived glioma xenograft), mouse anti-GAD67 (MAB5406, Millipore Sigma; Reactivity: Manufacturer validation: IF in mouse and rat hippocampi. Publication Figure: Extended Data Figure 6a, 6c, IF of patient-derived glioma xenograft), rabbit anti-H3K27M (ABE419, Millipore; Reactivity: mouse, human. Manufacturer validation: WB of lysates from MEF transfectants expressing FLAG-HA-tagged K27M histone H3.3. Publication Figure: Extended Data Figure 6c, IF of patient-derived glioma xenograft), mouse anti-cFos (sc-166940, Santa Cruz Biotechnology; Reactivity: Manufacturer validation: IF/ICC of SW480 cells and Hep G2 cells Publication Figure: Extended data figure 6d-e, IF of patient-derived glioma xenograft), chicken anti-Neurofilament-H (NFH, Aves Labs; Reactivity: human, mouse, rat, chicken, Manufacturer validation: IF rat cortical neurons and glia. Publication Figure: Figure 2c, Extended Data Figure 4a, IF of rat neuron/patient-derived glioma co-culture), chicken anti-Neurofilament-M (NFM, Aves Labs; Reactivity: human, mouse, rat, bovine, chicken. Manufacturer validation: ICC mouse cortical neurons. Publication Figure: Figure 2c, Extended Data Figure 4a, IF of rat neuron/patient-derived glioma co-culture), guinea pig anti-synapsin (106-004, Synaptic Systems; Reactivity: human, mouse, hamster, cow, zebrafish. Manufacturer validation: ICC immunostaining of PFA fixed rat hippocampus neurons. Publication Figure: Figure 2c, Extended Data Figure 4a, IF of rat neuron/patient-derived glioma co-culture), mouse anti-nestin (ab10538, Abcam; Reactivity: human, mouse. Manufacturer validation: IF human fetal neural progenitor cells, Publication Figure: Figure 2c, Extended Data Figure 4a, IF of rat neuron/patient-derived glioma co-culture), rabbit anti-GFP (NB600-308, Novus Biologicals; Reactivity: not species specific. Manufacturer validation: IF of GFP-expressing transgenic mouse pancreas. Publication Figure: Figure 2c, Extended Data Figure 4a, IF of rat neuron/patient-derived glioma co-culture).

Eukaryotic cell lines

Policy information about cell lines

Cell line source(s)	The eukaryotic cell cultures used are patient-derived cultures of high-grade gliomas generated in the Monje lab from biopsy (SU-pcGBM2) or autopsy tissue (SU-DIPG-VI, SU-DIPGXIII-FL, SU-DIPG36, SU-DIPG50, SU-DIPGXIII-P), or provided by collaborators from patient-derived cultures (SF0232, SF0238). For lentivirus generation, HEK293T cells were purchased from Thermo Fisher.
Authentication	Short Tandem Repeat (STR) fingerprinting is performed every 3 months on all cell cultures to ensure authenticity.
Mycoplasma contamination	All cell cultures are routinely tested for mycoplasma contamination and all cultures were tested negative.
Commonly misidentified lines<br>(See ICLAC register)	No commonly misidentified lines were used.

Animals and other organisms

Policy information about studies involving animals; ARRIVE guidelines recommended for reporting animal research

Laboratory animals	All animal experiments were conducted in accordance with protocols approved by the Stanford University Institutional Animal Care and Use Committee (IACUC) and performed in accordance with institutional guidelines. Animals were housed according to standard guidelines with free access to food and water in a 12 h light:12 h dark cycle, a temperature of 21°C, and 60% humidity. Housing temperature was XX and humidity was XX. For all xenograft studies, NSG mice (NOD-SCID-IL2R gamma chain-deficient, The Jackson Laboratory) aged 1-6 months were used. Male and female mice were used equally. For brain tumor xenograft experiments, the IACUC does not set a limit on maximal tumor volume but rather on indications of morbidity. In no experiments were these limits exceeded as mice were euthanized if they exhibited signs of neurological morbidity or if they lost 15% or more of their body weight. For neuron isolation, postnatal day 0 Sprague-Dawley rat pups of either sex were used.
Wild animals	No wild animals were used.
Field-collected samples	No field-collected samples were used.
Ethics oversight	All animal experiments were conducted in accordance with protocols approved by the Stanford University Institutional Animal Care and Use Committee (IACUC) and performed in accordance with institutional guidelines. The IACUC implements regulations from the United States Department of Agriculture (USDA), the Public Health Service (PHS) Policy, California State Regulations and Stanford University Polices and Guidelines to ensure effective and ethical animal research programs.

Note that full information on the approval of the study protocol must also be provided in the manuscript.

Human research participants

Policy information about studies involving human research participants

Population characteristics	Tissue from a single postmortem DMG case study was used.
Recruitment	N/A
Ethics oversight	For all human tissue and cell studies, informed consent was obtained, and tissue was used in accordance with protocols approved by the Stanford University Institutional Review Board (IRB).

Note that full information on the approval of the study protocol must also be provided in the manuscript.
